# GViT-GP: injecting the genomic relationship matrix as an inductive bias into a vision transformer via cross-attention for genomic prediction

**DOI:** 10.3389/fgene.2026.1758565

**Published:** 2026-03-09

**Authors:** Jingxuan Li, Wei Luo, Honghao Yu, Xishi Huang, Jisi Ma, Shijun Li, Yong Li, Lantao Gu

**Affiliations:** 1 College of Artificial Intelligence in Medicine, Guilin Medical University, Guilin, Guangxi, China; 2 College of Animal Sciences and Technology, Huazhong Agricultural University, Wuhan, Hubei, China

**Keywords:** cross-attention, deep learning, genomic prediction, genomic relationship matrix (GRM), genomic selection, inductive bias, vision transformer (ViT)

## Abstract

**Introduction:**

Genomic Prediction (GP) faces significant challenges in balancing model complexity with computational efficiency, particularly for high-dimensional genomic data under limited sample sizes.

**Methods:**

We propose GViT-GP, a Vision Transformer architecture that injects the Genomic Relationship Matrix (GRM) as a biological prior via a dual-pathway cross-attention fusion mechanism, coupled with a Selective Patch Embedding strategy to reduce redundancy and improve data efficiency.

**Results:**

We evaluated GViT-GP on 20 traits across four datasets from three species (soybean, cattle, and chicken). GViT-GP outperformed established linear and non-linear baselines (including GBLUP, LightGBM, and DNNGP), achieving the best accuracy in 16/20 tasks. Ablation studies supported the effectiveness of Selective Patch Embedding and cross-attention fusion, and visualization analyses suggest adaptive attention to informative genomic regions.

**Discussion:**

These results indicate that injecting GRM-informed inductive bias improves robustness and generalization in “p ≫ n” settings. GViT-GP provides a practical, high-performance framework for capturing complex genotype–phenotype relationships in modern digital breeding.

## 1 Introduction

Genomic prediction (GP), pioneered by Meuwissen et al. (2001), leverages genome-wide markers to forecast complex traits, thereby substantially accelerating modern breeding programs. The adoption of this methodology in U.S. Holstein cattle, for example, increased the annual rate of genetic gain by 50%–100% for milk production and three-to four-fold for low-heritability traits (García-Ruiz et al., 2016). This has effectively reoriented breeding strategies from a reliance on laborious phenotypic observation toward efficient, DNA-based early-life selection.

Classical GP methodologies, including Bayesian approaches and Best Linear Unbiased Prediction (BLUP) variants like GBLUP (VanRaden, 2008), are primarily predicated on an additive model assumption. While effective for many traits, this premise often limits the ability to capture non-additive effects and complex genetic architectures, where the phenotypic outcome is influenced by high-order interactions across the genome (Phillips, 2008). By design, the predictive power of these linear models is fundamentally constrained when facing such complexity.

In response to this limitation, researchers have explored more adaptive machine learning algorithms (Lourenço et al., 2024). Kernel-based methods, such as Support Vector Regression (SVR), employ the “kernel trick” to implicitly project SNPs into a higher-dimensional space to model non-linear relationships (Drucker et al., 1996; Long et al., 2011). Concurrently, ensemble models like LightGBM aggregate decision trees to capture interaction patterns (Ke et al., 2017; Yan et al., 2021). Yet, a shortcoming of these approaches is their treatment of SNPs as independent, position-agnostic features, thereby disregarding the inherent sequential structure of the genome (Zou et al., 2019).

Deep learning, particularly Convolutional Neural Networks (CNNs), provided new avenues by processing SNP sequences as one-dimensional signals. The sliding convolutional kernel is adept at capturing local dependencies between adjacent loci, an inductive bias exploited by models like DeepGS (Ma et al., 2017) and DNNGP (Wang et al., 2023). Nevertheless, the fixed and local receptive field of CNNs constrains their capacity to model the global dependencies and long-range patterns characteristic of complex traits (Vaswani et al., 2017; Dosovitskiy et al., 2020). To address this, some studies have sought to enhance the CNN architecture itself. For instance, the soyDNGP model (Gao et al., 2023) integrated a lightweight Coordinated Attention (CA) mechanism into the CNN backbone. This allows the model to capture broader spatial dependencies in a computationally efficient manner, representing a significant effort to overcome the locality constraint from within the convolutional framework.

The Transformer architecture’s self-attention mechanism, capable of modeling dependencies at arbitrary distances, is theoretically well-suited for capturing global genomic contexts (Vaswani et al., 2017). Its primary limitation, however, is the $O\left(n^{2}\right)$ computational complexity, which is often intractable for high-density SNP datasets. Prior work has sought to mitigate this issue through various means. GPTransformer (Jubair et al., 2021), for example, applied mutual information for feature selection. However, this metric typically focuses on univariate associations, potentially discarding markers that lack strong individual effects but are crucial for distinct predictive patterns. Similarly, GPformer (Wu et al., 2023) adopted an Auto-Correlation Attention mechanism to reduce complexity. While effective for forecasting tasks, this mechanism relies on identifying period-based dependencies in continuous time-series data. Applying such a temporal inductive bias to genomic data is suboptimal, as SNP sequences are discrete, spatial, and typically lack the inherent periodicity found in temporal signals.

In the field of Computer Vision, the Vision Transformer (ViT) has established a new state-of-the-art by demonstrating that high-dimensional data can be effectively modeled via a “patching” mechanism without sacrificing global context (Dosovitskiy et al., 2020). This insight allows for a drastic reduction in effective sequence length. However, directly applying standard image-based patching to ultra-high-dimensional genomic data can still result in noisy input representations or excessive computational load. Building upon the ViT paradigm, our work advances this approach by implementing a coarse-to-fine embedding strategy. Instead of indiscriminate dimensionality reduction, we first employ LightGBM to identify a non-linear, information-rich subset of loci, which effectively filters background noise. These high-value markers are then structured into patches, a design specifically engineered to resolve the computational bottleneck while maximizing the retention of critical genetic information. Furthermore, to address the risk of overfitting inherent in deep models under the classic “ p≫n ” scenario, we incorporate specific biological priors into the architecture.

To surmount these combined challenges, we propose GViT-GP, a ViT architecture engineered for genomic data. The design incorporates two key innovations:

- Selective Patch Embedding (SPE): This strategy refines the standard tokenization mechanism. By integrating the LightGBM-based pre-selection with patch embedding, we construct dense, information-rich tokens, ensuring computational efficiency while preserving loci with predictive potential to form complex patterns.
- Dual-Pathway Cross-Attention Fusion: To specifically contend with the overfitting prone “ p≫n ” problem, we introduce the Genomic Relationship Matrix (GRM) as a potent biological prior. A cross-attention mechanism serves as the fusion nexus, enabling the SNP representation (Query) to dynamically integrate population structure information from the GRM (Key/Value). This process injects a strong inductive bias, stabilizing the training process and guiding the ViT’s learning trajectory.

We validated GViT-GP on 20 distinct traits across four datasets. In a comparative benchmark against four established methods, including GBLUP and LightGBM, GViT-GP achieved superior predictive accuracy in 16 tasks and the lowest error in 17 tasks. These results substantiate its efficacy as a robust, next-generation framework for genomic prediction.

## 2 Materials and methods

### 2.1 Datasets

We utilized four datasets in this study:

Soybean (Soy15899): Sourced from the SoyBase database (Grant et al., 2010), this dataset comprises 15,899 samples genotyped with the SoySNP50K chip, for a total of 42,509 SNPs. Ten agronomic traits were recorded: protein content (protein), oil content (oil), linoleic acid content (Linoleic), linolenic acid content (Linolenic), days to flowering (R1), plant height (Hgt), days to maturity (Mat), lodging (Ldg), 100-seed weight (SdWgt), and yield (Yield).

Simulated Cows (Cows4020): This dataset, provided for the 16th QTLMAS Workshop in 2012 (Usai et al., 2014), is a simulated inbred population. It comprises 4,020 individuals, 9,969 SNPs, and three milk production-related traits (TA, TB, TC).

Holstein Bulls (Bulls1508): This dataset contains 1,508 Chinese Holstein bulls born between 1996 and 2016 (Yin et al., 2019). The samples were genotyped using the Illumina BovineSNP50K chip, yielding 44,074 SNPs. Five semen quality traits were recorded: sperm motility (SM), number of motile sperm per ejaculate (NMSP), total sperm number (NSP), sperm concentration (SC), and ejaculate volume (VE).

Chicken (Chicken192): Derived from our previous research (Luo et al., 2024), this dataset includes 192 Jinghong laying hens genotyped with the 600K Affymetrix Axiom HD array, resulting in 341,176 SNPs. Two reproductive traits were recorded: the sum of fertility days per fertilized egg after artificial insemination (DS) and the duration of fertilized egg production after a single insemination (DN).

All datasets underwent rigorous quality control (QC) using PLINK v1.9 (Purcell et al., 2007). SNPs were filtered based on the following criteria: call rate > 95%, minor allele frequency (MAF) > 0.01, and Hardy-Weinberg equilibrium (HWE) p-value $>1\times 10^{-6}$ . Following QC, each dataset was randomly partitioned into a training/validation set (80%) and a hold-out testing set (20%). The consistency of phenotypic distributions across these subsets was verified to ensure unbiased evaluation (e.g., for soybean R1 and sdWgt, see Figure 1; other traits are detailed in Supplementary Figures S1–S17). Each dataset can be accessed from the original paper.

**FIGURE 1:**
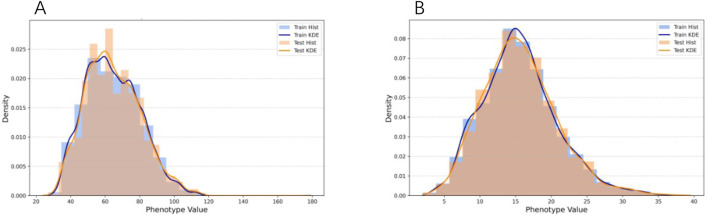
Phenotype distributions for the training and test sets. The plots display the density distributions of phenotype values, comparing the training set (blue) with the test set (orange). Both histograms and Kernel Density Estimation (KDE) curves are provided to demonstrate the consistent distribution between the two sets. **(A)** Distribution for the flowering time (R1) trait, measured in days. **(B)** Distribution for the hundred-seed weight (SdWgt) trait, measured in grams (g).

### 2.2 Overview of the model architecture

**FIGURE 2:**
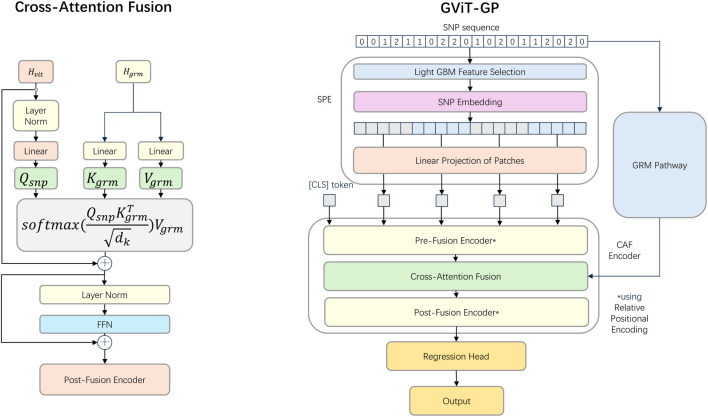
The overall architecture of GViT-GP and detailed schematic of the Cross-Attention Fusion module. The framework features a dual-pathway design: the Selective Patch Embedding (SPE) pathway processes raw SNP sequences via LightGBM-based selection and linear projection, while the parallel pathway encodes the Genomic Relationship Matrix (GRM). These representations converge in the Cross-Attention Fusion (CAF) Encoder, which comprises Pre-Fusion, Fusion, and Post-Fusion stages. Specifically, the Cross-Attention Fusion module utilizes the SNP representation $\left(H_{\mathrm{vit}}\right)$ to project the Query $\left(Q_{\mathrm{snp}}\right)$ , while the GRM representation $\left(H_{\mathrm{grm}}\right)$ serves as the Key $\left(K_{\mathrm{grm}}\right)$ and Value $\left(V_{\mathrm{grm}}\right)$ . This mechanism allows the model to dynamically inject population structure information as an inductive bias into the SNP features. The fused output is processed through residual connections, Layer Normalization, and a Feed-Forward Network, with the final prediction generated by a Regression Head using the [CLS] token.

The architecture of the proposed GViT-GP model is illustrated in Figure 2. It is a dual-pathway encoder composed of four primary components: a Selective Patch Embedding (SPE) module, a GRM pathway, a Cross-Attention Fusion (CAF) Encoder, and a regression head. The model first employs the SPE module to select a subset of informative SNPs. These SNPs are then converted into vector representations, partitioned into patches, and embedded via a linear projection. In parallel, the GRM for the population is pre-computed. One pathway processes the embedded SNP sequence, while the other processes the GRM. This information is then integrated within the CAF encoder via a cross-attention mechanism. Finally, a regression head utilizes the fused representation to predict the phenotype. The details of each module are elaborated upon below.

### 2.2.1 Selective Patch Embedding

The SPE module transforms the high-dimensional, raw SNP sequence into a low-dimensional, information-dense input. This design implements the “coarse-to-fine” strategy proposed in this study. First, to filter out background noise from the vast genomic search space, a LightGBM regression model (Ke et al., 2017) is employed as a coarse filter. We utilized the standard lightgbm Python package to calculate feature importance based on split gains, retaining only those SNPs with an importance score greater than zero.

Subsequently, the selected SNPs are organized into a sequence. To ensure consistent computational complexity across datasets with varying SNP densities, we adopted a fixed-patch-count strategy rather than a fixed-patch-size approach. Specifically, the sequence of selected SNPs (length L ) is partitioned into a fixed number of patches, $N_{p}$ . In our experiments, we set $N_{p}=400$ . This value was determined based on a preliminary sensitivity analysis testing $N_{p}\in \left\{200,400,800\right\}$ on a representative trait (Soybean Yield). We observed that $N_{p}=200$ resulted in underfitting due to coarse granularity. Interestingly, increasing the patch count to $N_{p}=800$ did not yield performance gains but rather led to a slight degradation in accuracy compared to $N_{p}=400$ , likely due to the increased difficulty in modeling global dependencies over longer sequences with limited sample sizes. Furthermore, $N_{p}=800$ significantly increased the computational burden. Therefore, $N_{p}=400$ was identified as the optimal configuration. To demonstrate the generalizability and potential of our framework, we fixed this hyperparameter across all datasets without conducting exhaustive, trait-specific tuning.Consequently, the patch size P is adaptively calculated as $P=\left\lceil L/N_{p}\right\rceil$ . If the total length L is not perfectly divisible by $N_{p}$ , zero-padding is applied to the final patch to maintain dimensional consistency. Each patch is then flattened and mapped to a D -dimensional embedding vector through a shared linear projection layer. This adaptive patching process aggregates local genetic information into a standardized sequence of compact tokens. Finally, a learnable classification (‘[CLS]’) token is prepended to the sequence of patch embeddings to serve as an aggregator for global features.

### 2.2.2 GRM pathway

**FIGURE 3:**
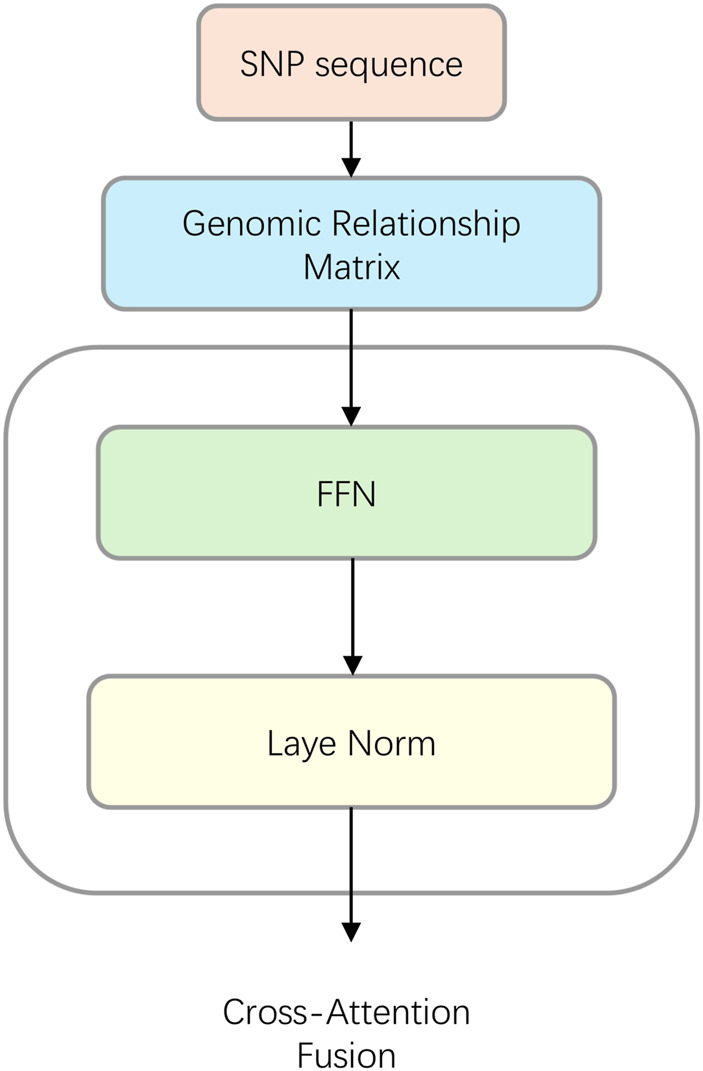
The architecture of the Genomic Relationship Matrix (GRM) pathway. From the full SNP sequence of all individuals, the GRM is calculated. For each individual, its corresponding vector from the GRM is fed into a Feed-Forward Network (FFN) and subsequently processed by a Layer Normalization block. The resulting representation serves as the Key and Value for the subsequent Cross-Attention Fusion module.

The GRM pathway (Figure 3) is designed to extract and represent the population structure and kinship information quantified by the Genomic Relationship Matrix (GRM). For each individual, the corresponding row vector from the GRM is fed into a feed-forward network (FFN). This FFN, consisting of two linear layers and a GELU activation function, projects the input GRM vector into a high-dimensional relationship representation, preparing it for the subsequent fusion step. The GELU activation is approximated as shown in Equation 1 (Hendrycks and Gimpel, 2016):

$$GELU\left(x\right)=0.5x\left(1+\tanh\left(\sqrt{\frac{2}{\pi }}\left(x+0.044715x^{3}\right)\right)\right)$$ (1)

The Genomic Relationship Matrix (GRM) is calculated using the VanRaden method (VanRaden, 2008) according to Equation 2:

$$G=\frac{M^{*}{\left(M^{*}\right)}^{\prime }}{2\sum_{j=1}^{m}p_{j}\left(1-p_{j}\right)}$$ (2)

where M is the marker genotype matrix with entries coded as 0, 1, or 2. The term $p_{j}$ is the frequency of the second allele at marker j , calculated as Equation 3:

$$p_{j}=\frac{\sum_{i=1}^{n}M_{ij}}{2n_{j}}$$ (3)

where $n_{j}$ is the number of individuals with a non-missing genotype at marker j . The genotype matrix M is centered to obtain $M^{*}=M-P$ , where P is a matrix in which every element in the j -th column is $2p_{j}$ .

### 2.2.3 Cross-attention fusion encoder

The core of GViT-GP is the Cross-Attention Fusion (CAF) Encoder, which integrates the processed feature embeddings from both pathways. The design of this module is motivated by a critical challenge: standard Transformers possess weak inductive biases, making them prone to overfitting on high-dimensional, small-sample-size (“ p≫n ”) genomic data. The GRM, in contrast, provides a strong biological prior regarding population structure and kinship. Therefore, we introduce a cross-attention mechanism to leverage the GRM as a dynamic inductive bias. This allows the model to guide the learning process on the SNP sequence using established biological relationships, thereby enhancing generalization.

**FIGURE 4:**
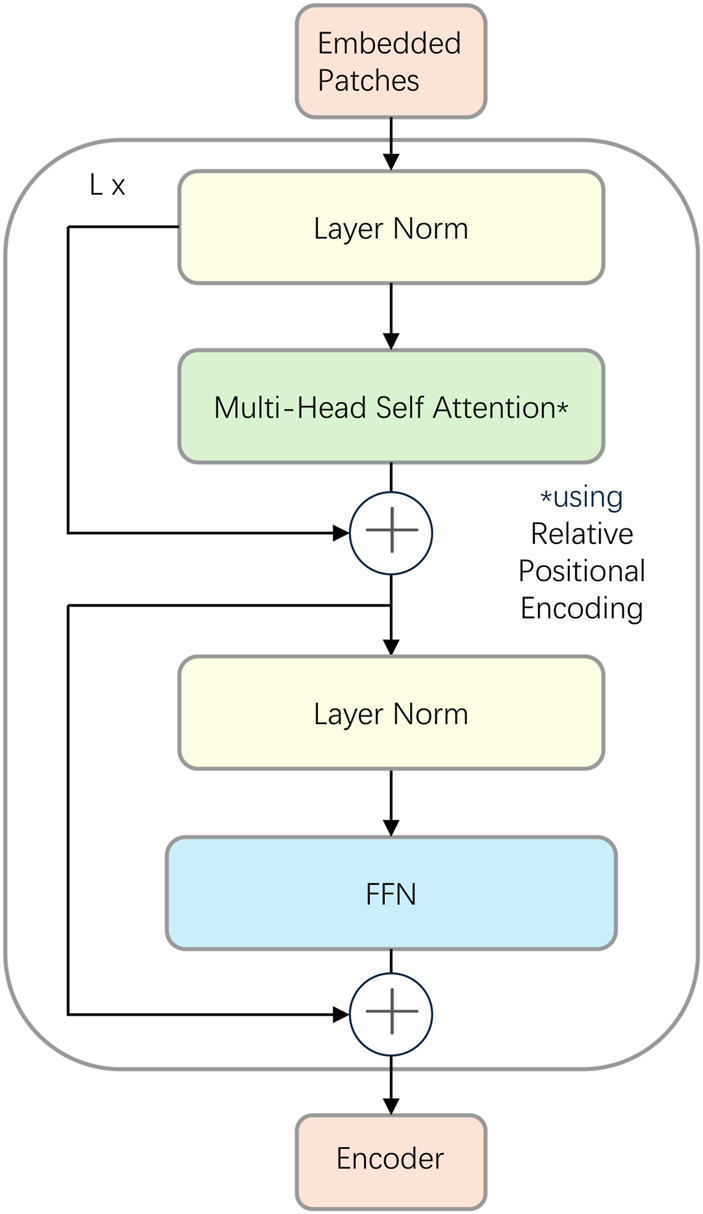
The architecture of the standard Transformer encoder layer used in GViT-GP. This diagram illustrates the Pre-Layer Normalization (Pre-LN) structure. The input (Embedded Patches) first passes through a Layer Normalization block, followed by a Multi-Head Self-Attention (MHSA) block which incorporates Relative Positional Encoding. A residual connection adds the output of the MHSA block to its input. This is followed by another sequence of Layer Normalization, a Feed-Forward Network (FFN), and a second residual connection. ‘L x’ denotes that this entire block is repeated L times.

The CAF Encoder consists of a stack of Transformer encoder layers, strategically divided by a central cross-attention module into two sub-components: a Pre-Fusion Encoder and a Post-Fusion Encoder. Crucially, each encoder layer adopts a Pre-Layer Normalization (Pre-LN) structure (Xiong et al., 2020). Unlike the standard Post-LN Transformer where normalization follows the residual block, Pre-LN applies Layer Normalization (LN) before the Multi-Head Self-Attention (MHSA) and Feed-Forward Network (FFN) blocks (Figure 4). This modification has been shown to improve gradient stability during backpropagation, enabling smoother convergence and preventing training instability often observed in deep networks on sensitive datasets.

The computation for the l -th Pre-LN encoder block is defined as Equation 4, and the head of MHSA is defined as Equation 5:

$$\begin{array}{cc} x_{l}^{\prime } & =x_{l}+\text{MHSA}\left(\text{LN}\left(x_{l}\right)\right)\\ x_{l+1} & =x_{l}^{\prime }+\text{FFN}\left(\text{LN}\left(x_{l}^{\prime }\right)\right) \end{array}$$ (4)

where $x_{l}$ denotes the input to the layer, $x_{l}^{\prime }$ is the intermediate representation, and $x_{l+1}$ is the output.

$${\text{head}}_{i}=\text{Attention}\left(Q_{i},K_{i},V_{i}\right)=\text{softmax}\left(\frac{Q_{i}K_{i}^{T}}{\sqrt{d_{k}}}+B_{i}\right)V_{i}$$ (5)

The bias matrix $B_{i}$ provides positional information, where each element ${\left(B_{i}\right)}_{j,k}$ is retrieved from a learnable lookup table using the relative position k−j .

We used a learnable relative positional bias (indexed by relative distance k−j ) that is added to the self-attention logits in every Transformer encoder block, following the form in 5. This positional encoding scheme was kept identical across all ablation variants to ensure a fair comparison. The cross-attention fusion module does not introduce an additional positional term because its keys/values originate from the GRM pathway rather than an ordered genomic token sequence.

The cross-attention fusion module serves as the fusion nexus. It is a multi-head attention layer with an asymmetric input structure: the query (Q) is derived from the SNP pathway representation $\left(H_{\mathrm{snp}}\right)$ , while the key (K) and value (V) are derived from the GRM pathway representation $\left(H_{\mathrm{grm}}\right)$ . This operation is expressed as shown in Equations 6, 7:

$$\left\{\begin{array}{cc} Q_{\mathrm{snp}} & =\;H_{\mathrm{snp}}W_{i}^{Q}\\ K_{\mathrm{grm}} & =\;H_{\mathrm{grm}}W_{i}^{K}\\ V_{\mathrm{grm}} & =\;H_{\mathrm{grm}}W_{i}^{V} \end{array}\right.$$ (6)

$$\text{Attention}\left(Q_{\mathrm{snp}},K_{\mathrm{grm}},V_{\mathrm{grm}}\right)=\text{softmax}\left(\frac{Q_{\mathrm{snp}}K_{\mathrm{grm}}^{T}}{\sqrt{d_{k}}}\right)V_{\mathrm{grm}}$$ (7)

This design ensures that as SNP representations pass through the cross-attention module, they actively query and integrate population structure information encoded in the GRM. This mechanism facilitates a dynamic fusion of genomic features with relational priors, effectively regularizing the model and mitigating the risk of overfitting.

### 2.2.4 Regression head

The final hidden state corresponding to the ‘[CLS]’ token, which serves as an aggregated representation of the input sequence, is passed to the regression head. The regression head is a multi-layer perceptron (MLP) composed of linear layers, an activation function, and a dropout layer for regularization, which maps the final representation to a scalar phenotype value.

### 2.3 Experimental design and evaluation

To ensure a robust and unbiased assessment, our evaluation protocol strictly partitioned each dataset into an 80% training/validation set and a 20% independent hold-out test set. Within the training phase, hyperparameters were optimized using a 5-fold cross-validation procedure, while the final model performance was reported based on a single evaluation on the unseen test set.To prevent data leakage, locus selection was nested within the cross-validation loop. Specifically, for each of the five folds, the selector (LightGBM/linear SVR/GWAS + LD) was trained using only the fold-specific training partition, and the selected loci were then used to transform both the training and validation individuals of that fold. The held-out test set (20%) was never used for locus selection, hyperparameter tuning, or early stopping. After model selection, we refit the selector and the downstream model on the full training/validation split (80%) and evaluated once on the untouched test set. To avoid data leakage, GRM features were constructed in a fold-aware manner. For each cross-validation fold, allele frequencies and marker centering were estimated using only the fold-specific training partition. We then computed (i) the within-training GRM for training individuals $\left(G_{\text{train}\times train}\right)$ , and (ii) for each validation individual, its relationship vector with respect to the training cohort $\left(G_{\text{val}\times train}\right)$ , which was used as the GRM-pathway input. For the final evaluation, we estimated allele frequencies on the full training/validation split (80%) and computed the relationship vector for each held-out test individual with respect to this cohort $\left(G_{\text{test}\times train}\right)$ . The test set was never used to estimate allele frequencies or to construct training-time GRM features.

We benchmarked GViT-GP against four established methods representing diverse algorithmic classes. For the linear baseline, we implemented GBLUP using the standard R package rrBLUP (Endelman, 2011) to guarantee the reliability of the genomic relationship matrix implementation. Non-linear machine learning baselines included Support Vector Regression (SVR) with an RBF kernel (implemented in scikit-learn) and LightGBM, a gradient boosting decision tree method implemented via its official Python package (Ke et al., 2017). Additionally, we compared our approach with DNNGP (Wang et al., 2023), a state-of-the-art CNN-based deep learning model using its official PyTorch release.

All deep learning models were implemented in PyTorch v2.4.0 (Paszke et al., 2019). Training was conducted to minimize the Mean Squared Error (MSE) loss using the AdamW optimizer (Loshchilov and Hutter, 2017), stabilized by a cosine annealing learning rate scheduler (Loshchilov and Hutter, 2016) and an early stopping mechanism to prevent overfitting. Model efficacy was quantified using three standard metrics: Mean Squared Error (MSE), Pearson correlation coefficient (r), and the coefficient of determination $\left(R^{2}\right)$ .

### 2.4 Ablation studies

Three targeted ablation studies were conducted to dissect the GViT-GP architecture. Collectively, they address (i) the trade-off between computational efficiency and information integrity in SNP tokenization, (ii) how inductive bias is introduced through GRM integration, and (iii) how the upstream locus-selection strategy influences the downstream Transformer’s predictive performance.

The first study investigated the optimal tokenization strategy for SNP sequences. We sought to isolate the benefits of our proposed “coarse-to-fine” approach by comparing three configurations on an identical ViT backbone: (1) Full-sequence Patch Embedding (FPE), which partitions the entire unfiltered SNP sequence; (2) Selective Independent Embedding (SIE), a baseline that treats the selected SNPs as independent tokens without patching; and (3) our proposed Selective Patch Embedding (SPE), which aggregates information-rich SNPs into local patches to maximize context retention while reducing sequence length.

The second study evaluated the hypothesis that dynamically integrating the GRM via cross-attention offers a superior inductive bias compared to static fusion. We contrasted single-pathway baselines (GViT-Base and GRM-MLP) against a naive Static Fusion (GViT-Concat) approach, where features from both pathways are simply concatenated. These were compared with the proposed Dynamic Fusion (GViT-GP) model. By employing the cross-attention mechanism, GViT-GP enables the SNP representation to actively query population-structure information encoded in the GRM, providing a more robust and context-aware biological prior.

The third study examined the impact of the locus-selection strategy used to construct the informative SNP subset. Specifically, we compared the LightGBM-based selector against two established alternatives: a multivariate linear selector based on linear-kernel SVR and a univariate GWAS-based filtering pipeline with LD pruning (GWAS + LD). For the GWAS + LD baseline, to mitigate multicollinearity, We performed LD pruning using PLINK v1.9 with a 200 kb window size, a step size of 1, and an $r^{2}$ threshold of 0.5 To eliminate confounding effects arising from varying input dimensionalities, we aligned the feature count across all methods. Specifically, the number of loci retained (k) for the baseline selectors was set to strictly match the number of informative features identified by the LightGBM model (i.e., those with non-zero importance scores). We did not use a fixed p -value cutoff; instead, we retained the top- k SNPs with the smallest GWAS p -values after LD pruning. This ablation isolates whether the advantage of GViT-GP stems merely from dimensionality reduction or from the selector’s ability to prioritize jointly informative loci for subsequent Transformer modeling. Unless otherwise stated, locus selection was performed using the training split only to prevent data leakage.

## 3 Results

### 3.1 Impact of embedding strategy on GViT-GP

**FIGURE 5:**
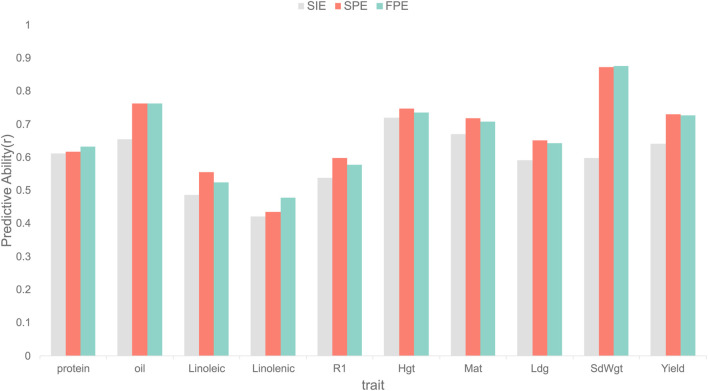
Comparison of predictive ability for three different embedding strategies on soybean agronomic traits. The y-axis represents the predictive ability, measured as the Pearson correlation coefficient (r). Three strategies are compared: Selective Independent Embedding (SIE), Selective Patch Embedding (SPE), and Full-sequence Patch Embedding (FPE). The comparison is performed across ten different traits.

Our investigation into embedding strategies confirms that the tokenization, the core concept of the Vision Transformer architecture, successfully translates its effectiveness to the domain of genomic prediction. As illustrated in Figure 5, a stark performance gap exists between the two strategies that employ tokenization (SPE and FPE) and the non-ViT baseline that does not (SIE). The SIE strategy, which processes individually selected SNPs as independent tokens, demonstrated markedly inferior predictive ability across all ten agronomic traits. This result strongly indicates that preserving local sequence information by grouping SNPs into “patches” is a fundamental prerequisite for the model to learn meaningful genetic patterns, and that a standard Transformer applied to a simple list of important features is insufficient.

Building on the confirmed viability of this tokenization-based strategies, we then sought to identify its optimal implementation. The comparison between the naive application (FPE) and our proposed selective approach (SPE) was nuanced, but ultimately favored SPE as the more robust strategy. The results revealed a competitive landscape: SPE achieved higher prediction accuracy (r) on a majority of traits (six out of ten), while FPE held a slight advantage on the remaining four. For the traits where SPE excelled (Linoleic, R1, Hgt, Mat, Ldg, and Yield), it offered modest but consistent performance gains, with r-value improvements of 0.0304, 0.0211, 0.0120, 0.0112, 0.0090, and 0.0031, respectively, over FPE. Conversely, on the four traits where FPE was superior (protein, oil, linolenic, and SdWgt), SPE’s performance was only marginally lower, with the respective differences being 0.0153, 0.0001, 0.0435, and 0.0040. Supplementary Table S1 shows the detailed data. Although the margin was narrow, this outcome suggests that the SPE strategy strikes a more effective balance. Its ability to perform best on a majority of traits, coupled with the fact that its losses were marginal, points to its greater robustness across diverse genetic architectures. Its superior performance on a majority of traits indicates that pre-selecting for information-dense regions is a beneficial adaptation, leading to a more robust and consistently performing genomic ViT model.

### 3.2 Impact of fusion mechanism on GViT-GP

Table 1 summarizes the predictive performance of the four model variants, averaged across all 10 soybean agronomic traits. Detailed results for individual traits are provided in Supplementary Table S2. The aggregated results clearly demonstrate that the proposed GViT-GP model significantly outperformed the other three variants across all evaluation metrics. Specifically, compared to the simple feature concatenation strategy (GViT-Concat), GViT-GP’s mean prediction accuracy (r) was 0.0384 higher, demonstrating that cross-attention is a superior fusion method for integrating multi-source information.

**TABLE 1:** Average predictive performance of four model variants in the soybean dataset.

Model	r (avg)	R2 (avg)	MSE (avg)
GViT-Base	0.668615	0.448868	0.560036
GRM-MLP	0.689772	0.482608	0.523069
GViT-Concat	0.68393	0.47764	0.529607
GViT-GP	**0.72235**	**0.52727**	**0.473195**

Supplementary Table S2 shows the detailed data. Bold values indicate the best performance among the compared model variants (highest r and R^2^; lowest MSE).

Furthermore, GViT-GP’s performance surpassed that of its constituent pathways when evaluated in isolation, increasing the average prediction accuracy (r) by 0.0537 and 0.0326 compared to the stand-alone GViT-Base and GRM-MLP models, respectively. This result strongly indicates that the cross-attention framework facilitates a synergistic effect, effectively leveraging the complementary information from both the SNP and GRM pathways to improve overall model performance.

### 3.3 Impact of locus-selection strategy on GViT-GP

**FIGURE 6:**
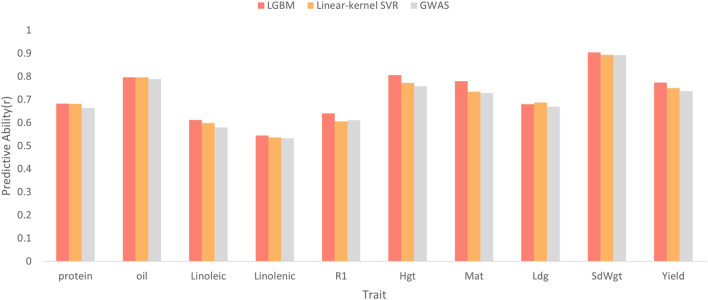
Comparison of predictive performance across ten soybean traits using different locus-selection strategies.The bar chart displays the predictive ability (measured by Pearson’s correlation coefficient, r ) for the proposed LightGBM-based selector compared to two baselines: Linear-kernel SVR and GWAS with LD pruning. The x-axis lists the ten target traits, and the y-axis represents the predictive accuracy. LightGBM consistently outperforms the baselines, achieving the highest accuracy in eight out of 10 traits. Notable improvements are observed in complex traits such as Yield and Linoleic acid, indicating the method’s effectiveness in capturing non-linear relationships and joint informativeness compared to linear and univariate approaches.

To evaluate the effectiveness of the proposed feature selection module, we compared our LightGBM-based strategy against two established baselines: a linear-kernel SVR and GWAS-based filtering with LD pruning (GWAS + LD). The comparative results across ten soybean traits are summarized in Figure 6.

Overall, the LightGBM strategy demonstrated robust performance, achieving the highest predictive accuracy on eight out of 10 traits. Notably, its advantage was more pronounced for traits with potentially more complex genetic architectures. For example, in yield (Yield) prediction, LightGBM attained a correlation of 0.7743, corresponding to absolute improvements of +0.0242 and +0.0376 over linear SVR (0.7501) and GWAS + LD (0.7367), respectively. Similarly, for linoleic acid (Linoleic acid), LightGBM improved correlation by approximately +0.013 and +0.032 compared to linear SVR and GWAS + LD.

While linear SVR remained competitive on a few traits (e.g., Oil and Lodging/Ldg), it did not surpass LightGBM on most targets; in contrast, GWAS + LD generally resulted in the lowest predictive performance. Taken together, these observations suggest that a multivariate selector based on gradient-boosted decision trees can more effectively leverage jointly informative loci and is better suited to capturing non-linear relationships and feature interactions, thereby providing a higher-information SNP subset for subsequent Transformer-based modeling.

In addition, we adopted LightGBM as the locus selector for three practical considerations. First, its modeling capacity for non-linearity and feature interactions helps retain loci with stronger joint informativeness at the selection stage. Second, LightGBM is computationally efficient and scalable in large-SNP settings, enabling stable selection within a reasonable time budget. Third, we determine the retained loci based on whether feature importance is non-zero, which avoids introducing trait-specific threshold tuning (e.g., GWAS p -value cutoffs) and allows us to include all loci that contribute measurable information, aligning with our end-to-end modeling philosophy that minimizes heuristic, trait-dependent interventions.

Finally, to avoid confounding due to different input dimensionalities, both linear SVR and GWAS + LD were constrained to select the same number of loci as LightGBM (a fixed- k controlled setting). We did not further tune method-specific optimal k values; therefore, the results should be interpreted as a dimension-matched comparison of selection strategies rather than each baseline’s best achievable performance. We also note that the fixed- k protocol may force certain methods to include noisier loci.

### 3.4 Comparative analysis of GViT-GP with other GP models

**FIGURE 7:**
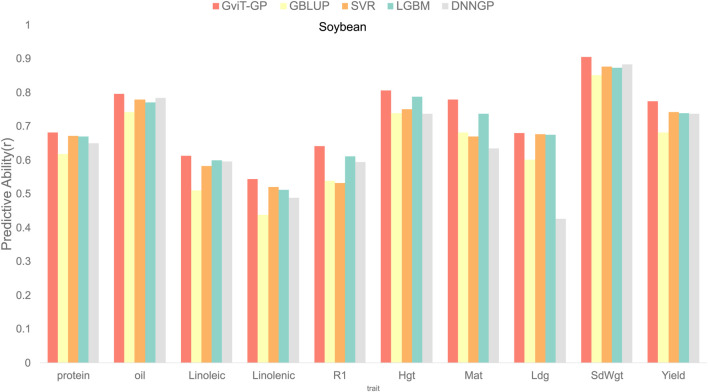
Comparison of predictive ability between GViT-GP and baseline models on the soybean dataset. The bar chart illustrates the performance, measured by the Pearson correlation coefficient (r) as Predictive Ability, of GViT-GP against four established baseline models: GBLUP, Support Vector Regression (SVR), LightGBM, and DNNGP. The evaluation was conducted across ten key agronomic traits of the soybean dataset.

First, we compared GViT-GP against four baseline models on the soybean dataset, which includes 10 key agronomic traits (Figure 7). The results revealed a significant performance advantage for GViT-GP. In terms of prediction accuracy (r), GViT-GP was the top-performing model on all traits, outperforming the next-best model by a margin of 0.0028–0.0318. It also achieved the highest coefficient of determination $\left(R^{2}\right)$ in nine of the traits. Supplementary Table S3 shows the detailed data.

**FIGURE 8:**
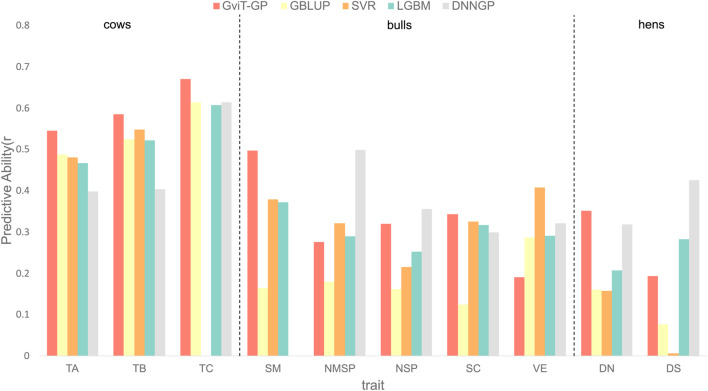
Comparison of predictive ability across different models on three animal datasets. The bar chart displays the Pearson correlation coefficient (r), termed Predictive Ability, for GViT-GP and four baseline models: GBLUP, Support Vector Regression (SVR), LightGBM (LGBM), and DNNGP. The comparison is conducted across ten traits from three animal datasets (two cattle cohorts: simulated cows and Holstein bulls; one chicken cohort: hens): simulated cows (TA, TB, TC), Holstein bulls (SM, NMSP, NSP, SC, VE), and hens (DN, DS).

To further assess the model’s generalization ability, we evaluated it on three animal datasets characterized by smaller sample sizes and more diverse genetic architectures (Figure 8). Across a total of 10 animal traits, GViT-GP achieved the highest prediction accuracy (r) in six traits, with improvements of 0.0183–0.1188 over the next-best model. It also obtained the highest $R^{2}$ in eight traits, with improvements of 0.0228–0.3701 (see Supplementary Material for details). Supplementary Tables S4–S6 shows the detailed data. Notably, the DNNGP model yielded non-predictive results (e.g., negative $R^{2}$ ) for the SM trait and underperformed traditional machine learning models on several other traits. In contrast, GViT-GP maintained robust performance across all tasks, highlighting its architectural advantages.

**FIGURE 9:**
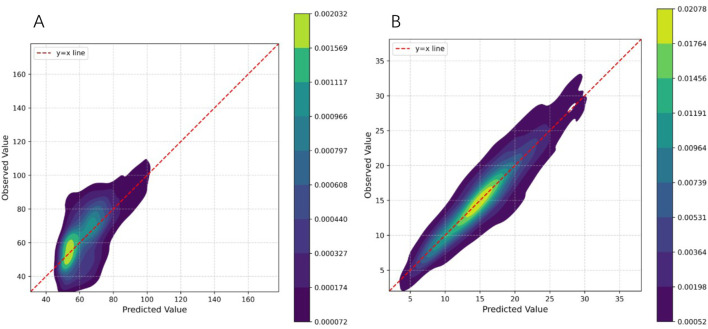
Two-dimensional kernel density plots of predicted versus observed values. The x-axis represents the values predicted by GViT-GP, and the y-axis represents the true observed values. The color intensity corresponds to the density of data points. The dashed red line represents the y = x line, indicating a perfect prediction. **(A)** Plot for the flowering time (R1) trait. **(B)** Plot for the hundred-seed weight (SdWgt) trait.

To more intuitively assess the model’s predictive performance, we plotted 2D kernel density maps of the predicted versus observed values (Figure 9). Taking the key agronomic traits of R2 and SdWgt in the soybean dataset as exame, these plots provide a granular view of the model’s predictive behavior. For the more genetically complex R2 trait (Figure 9A), while the distribution is broader, the core density remains tightly clustered along the diagonal, demonstrating that the model successfully captured the primary genetic signal of the trait. Conversely, for SdWgt (Figure 9B), the density of data points is highly concentrated along the y = x diagonal in a tight, narrow distribution, indicating minimal prediction error and high consistency between predicted and true values. Collectively, these plots confirm that GViT-GP not only excels on aggregate metrics but also generates predictions with no discernible systemic bias, showing a close linear correspondence with true phenotypic values. Similar plots for other traits are provided in Supplementary Figures S18–S24.

In summary, across all 20 traits evaluated, GViT-GP achieved the highest prediction accuracy (r) in 16 tasks and the highest coefficient of determination $\left(R^{2}\right)$ in 17 tasks. These results fully demonstrate the strong potential for GViT-GP’s application across different species and genetic structures.

### 3.5 Generalization performance on external validation set

A critical challenge in genomic prediction is the transferability of models across different populations or genetic backgrounds. To rigorously assess the practical robustness of GViT-GP beyond the initial training population, we conducted an external validation using a distinct bi-parental population from the Soybean Nested Association Mapping (SoyNAM) project (Diers et al., 2018). Specifically, we utilized the NAM03 population, which is derived from the cross involving the specific parent 4J105-3-4. Validation on such a structured NAM family serves as a stringent test to evaluate whether the model captures intrinsic biological signals that persist across distinct genetic lineages, rather than merely memorizing the population structure of the training set.

**FIGURE 10:**
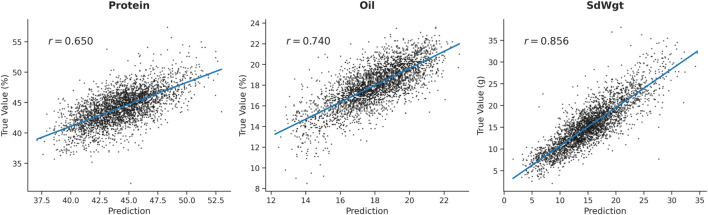
External validation of GViT-GP on the independent SoyNAM-03 population (Parent: 4J105-3-4). The scatter plots illustrate the relationship between the predicted phenotypic values (x-axis) and the true observed values (y-axis) for three representative traits: Protein, Oil, and Hundred-Seed Weight (SdWgt). The blue line represents the linear regression fit. The Pearson correlation coefficient (r) is annotated for each trait, demonstrating strong generalization capability.

As illustrated in Figure 10, GViT-GP demonstrated strong generalization capabilities on this external cohort. The scatter plots reveal a strong linear correspondence between the predicted and observed values across the evaluated traits. Specifically, the model achieved a high predictive correlation of r=0.856 for the Hundred-Seed Weight (SdWgt) trait, indicating high robustness in capturing the genetic architecture of yield-related traits across populations. Similarly, the Oil and Protein traits maintained strong predictive performance with correlations of r=0.740 and r=0.650 , respectively.

These results are particularly significant given that the model was frozen after training and directly applied to the specific 4J105-three to four derived family without fine-tuning. The ability to maintain the accuracy on a genetically distinct sub-population confirms that GViT-GP effectively mitigates the risk of overfitting and successfully extracts genomic features, satisfying a key requirement for practical breeding deployment.

### 3.6 Interpretability analysis

**FIGURE 11:**
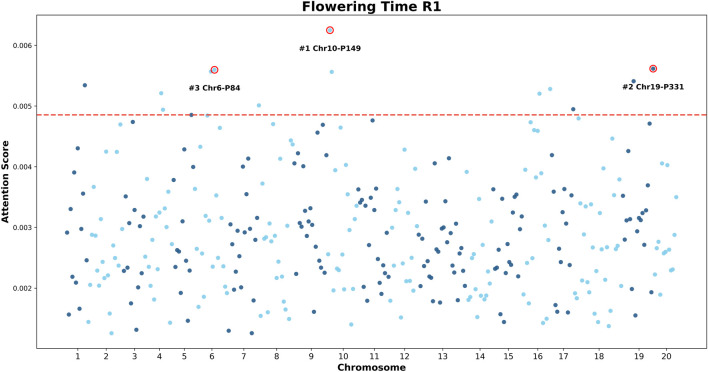
Manhattan plot of attention scores for the soybean flowering time (R1) trait. The x-axis represents the genomic position (chromosome number), and the y-axis represents the attention score of the [CLS] token assigned to each SNP patch. The red dashed line indicates the significance threshold. Key peaks are annotated, identifying high-attention patches on Chromosomes 6, 10, and 19. Notably, the peak on Chromosome 6 (Patch #3, Chr6-P84) corresponds to a known quantitative trait locus (QTL) region.

To investigate whether the GViT-GP model captures biologically meaningful genomic signals rather than merely fitting statistical artifacts, we visualized model interpretability by mapping the attention weights of the [CLS] token back to physical genomic coordinates. Figure 11 presents “Manhattan-like” plots for soybean flowering time (R1) and seed oil content (Oil), where the x-axis represents the chromosomal position of SNP patches and the y-axis denotes the attention score assigned by the model.

As shown in Figure 11, the attention distribution for the R1 trait is distinctly non-uniform, exhibiting clear peaks at specific loci. The model autonomously identified several high-attention regions, most notably a significant peak on Chromosome 6 (labeled #3 Chr6-P84), as well as peaks on Chromosomes 10 and 19. This concentrated attention pattern aligns with the genetic architecture of R1, which is known to be governed by major-effect loci such as the *E1* gene located in this region of Chromosome 6.

**FIGURE 12:**
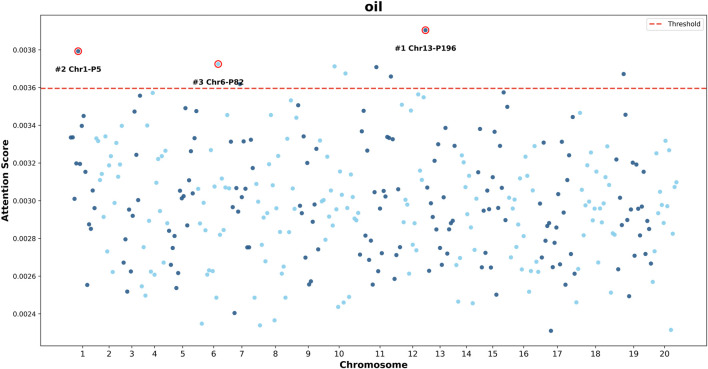
Manhattan plot of attention scores for the soybean seed oil content (oil) trait. The x-axis represents the genomic position (chromosome number), and the y-axis represents the attention score of the [CLS] token assigned to each SNP patch. The red dashed line indicates the significance threshold. Key peaks are annotated, identifying high-attention patches on Chromosomes 13, 1, and 6. Notably, the peak on Chromosome 6 (Patch #3, Chr6-P82) lies within (or in close proximity to) the reported qOIL-3 region, which contains the candidate gene *Glyma06g20900* (*Glyma.06G194500* in Wm82.a2) implicated in seed lipid metabolism.

To further validate the model’s adaptability to diverse genetic architectures, we extended the analysis to the Oil trait (Figure 12). Unlike R1, oil content is typically characterized as a complex, polygenic trait regulated by numerous loci with small-to-moderate effects. This biological distinction is clearly reflected in the attention maps generated by GViT-GP. The attention distribution for Oil also displays significant non-uniformity. The most prominent peak is located on Chromosome 13 (#1 Chr13-P196), followed by Chromosome 1 (#2 Chr1-P5) and Chromosome 6 (#3 Chr6-P82), while the vast majority of patches form a relatively tight background band. It is important to emphasize that the y-axis ranges for the R1 and Oil attention plots are not identical. In the current visualization, the attention scores for R1 span a larger range (extending from ∼ 0.001 to over 0.006), whereas the scores for Oil are displayed within a much narrower interval (approximately ∼ 0.0023 to ∼ 0.0039). This discrepancy aligns with biological expectations: the flowering time trait (R1) often involves stronger major-effect signals, resulting in higher contrast in attention scores, whereas oil content is typically co-regulated by more small-effect loci, leading to a more “compressed” distribution of attention scores.

To assess whether the interpretability patterns generalize beyond the exemplar cases shown in the main text, we provide attention heatmaps for the remaining soybean traits as well as an additional attention heatmap on the Bulls1508 dataset in the Supplementary Material (Supplementary Figures S24–S34), which are included for qualitative comparison of structured, non-uniform attention patterns without trait-specific biological interpretation.

## 4 Discussion

The proposed GViT-GP, by resolving the conflict between the complexity of Vision Transformers and the constraints of genomic data, has demonstrated robust performance across 20 traits in four datasets. This work provides a novel and viable framework for effectively modeling complex genetic architectures using deep learning. Our ablation studies offer clear explanations for the internal mechanisms of this design, validating the efficacy of the “coarse-to-fine” embedding and the dual-pathway fusion. The high consistency between predicted and observed values in this study, especially as demonstrated in the density plots for soybean 100-seed weight and yield traits, provides deeper evidence for the effectiveness of our framework. This close fit, particularly in the absence of systemic over- or under-estimation, strongly indicates that GViT-GP is not only statistically accurate but has also learned a robust mapping from genotype to phenotype.

Our work first addresses the fundamental question of whether the image-centric Vision Transformer (ViT) paradigm can be applied to genomic sequences. Our ablation studies provide a definitive answer, demonstrating that strategies utilizing tokenization (SPE and FPE) outperform the non-tokenization strategy (SIE). This comparison reveals that preserving the local context of SNPs through tokenization is crucial for genomic prediction. This finding establishes that the core mechanism of ViT—aggregating local information into global representations—is highly suitable for this task. Among the tokenization-based strategies, we observed that SPE is the more robust approach. This suggests that in the high-dimension, low-sample-size (p≫n) context, this strategy effectively filters out substantial genomic background noise prior to data entry into the Transformer. By employing LightGBM for feature selection, we retained a maximal number of informative SNPs, thereby obtaining an input set with a high signal-to-noise ratio. Moreover, SPE enhances the model’s resolution under an identical computational budget, enabling a finer-grained representation of details. This indicates that, compared to the theoretically more comprehensive but computationally demanding FPE, the SPE strategy represents a superior engineering choice under realistic constraints.

While conventional strategies for injecting inductive bias, such as pre-training large-scale models (e.g., DNAbert (Ji et al., 2021)), are powerful, their application in quantitative genetics is often hindered by platform-specific markers and prohibitive costs. In contrast, our proposed GRM solution is computationally friendly and requires no extra data, reflecting its value as a pragmatic source of inductive bias. Furthermore, we demonstrated that simple feature concatenation (GViT-Concat) is insufficient for fusing features with disparate inductive biases. We chose the cross-attention mechanism because it aligns with our hypothesis: the population structure provided by the GRM should not have a uniform, static influence on all SNP patches. Cross-attention allows the SNP pathway to dynamically query and borrow kinship information from the GRM, providing precise “learning guidance.” Its robustness was validated even on traits where DNNGP failed.

Crucially, the practical value of a genomic prediction model hinges on its cross-population generalization capability. Our external validation on the independent NAM03 population (derived from the specific parent 4J105-3-4 (Diers et al., 2018)) demonstrated that GViT-GP maintains high predictive accuracy (e.g., r=0.856 for SdWgt) without any fine-tuning. This result is pivotal, as it counters the common concern that deep learning models tend to overfit the specific population structure of the training set. Instead, it suggests that GViT-GP has successfully captured transferable, intrinsic biological signals, satisfying a key requirement for practical breeding deployment where target populations often differ from training cohorts.

Beyond predictive accuracy, the attention mechanism in GViT-GP challenges the notion that deep learning models are opaque or lack interpretability. A compelling validation of this capability is provided by our attention-to-genome mapping analyses on soybean traits. For flowering time (R1), the attention Manhattan plot (Figure 11) shows that GViT-GP autonomously highlights a high-confidence region on Chromosome 6. Detailed examination reveals that the high-attention patch encompasses SNPs (e.g., ss715593840) located in the proximal region of *Glyma06g23026*, which corresponds to *E1* (Xia et al., 2012), a major dominant regulator of flowering time and maturity in soybean (Zhai et al., 2022). Although this SNP does not reside within the coding sequence, its detection strongly suggests that GViT-GP captured the underlying causal signal via linkage disequilibrium (LD), thereby focusing on a verified QTL locus. Importantly, a consistent interpretability pattern is also observed for seed oil content: the oil attention Manhattan plot highlights a peak on Chromosome six overlapping the reported qOIL-3 region. Notably, this interval contains *Glyma06g20900* (corresponding to *Glyma.06G194500* in Wm82.a2), which encodes a GDSL-motif lipase/hydrolase and has been proposed as a candidate gene linked to seed lipid metabolism and oil-content regulation (Huynh et al., 2024). Collectively, these results indicate that the model’s learned representations are not merely statistical artifacts but align with biologically meaningful loci, supporting the use of GViT-GP as a hypothesis-generation tool for prioritizing functional genomic regions.

Despite the promising results, this study has limitations that open avenues for future research. The primary limitation lies in the current two-stage implementation of SPE, which relies on LightGBM. While LightGBM is efficient, as a tree-based method, it naturally favors SNPs with strong main effects, potentially filtering out loci that are critical only within complex, high-order interaction networks but have weak individual effects (Strobl et al., 2008). This implies that the current bias is directed towards capturing additive genetic signals, potentially under-representing complex non-additive (e.g., epistatic) variations. This motivates the future exploration of end-to-end differentiable frameworks to overcome this feature selection bottleneck. Another consideration is the computational cost. We conducted a detailed analysis of training time versus performance (Supplementary Table S7). Although GViT-GP requires more training time (1.63 h) compared to traditional methods (e.g., 1 min for LightGBM), this increase is negligible in the context of practical breeding cycles, which span months or years. The additional computational overhead is a justifiable one-time offline investment that yields significant improvements in predictive accuracy. To further address these demands, future optimization efforts could explore efficient attention variants (e.g., Perceiver or Linear Attention) or model distillation techniques to facilitate rapid deployment.

In conclusion, the GViT-GP architecture offers an effective approach to adapting Vision Transformers for genomic prediction. By integrating a SPE strategy and a GRM-informed inductive bias, it presents a robust framework for modeling quantitative traits. The consistent performance observed across diverse species, combined with the demonstrated cross-population generalization and biological interpretability, highlights the potential of GViT-GP as a promising tool to support modern digital breeding programs.

## Data Availability

The original contributions presented in the study are included in the article/Supplementary Material, further inquiries can be directed to the corresponding authors.
