# Supplementary material for: GViT-GP: injecting the genomic relationship matrix as an inductive bias into a vision transformer via cross-attention for genomic prediction
Source: Front Genet. 2026 Mar 9;17:1758565. doi: 10.3389/fgene.2026.1758565 (PMC13006091; doi:10.3389/fgene.2026.1758565)
Supplement: Supplementary file 8 [file Image1.pdf]

Supplementary Material

1 SUPPLEMENTARY FIGURES

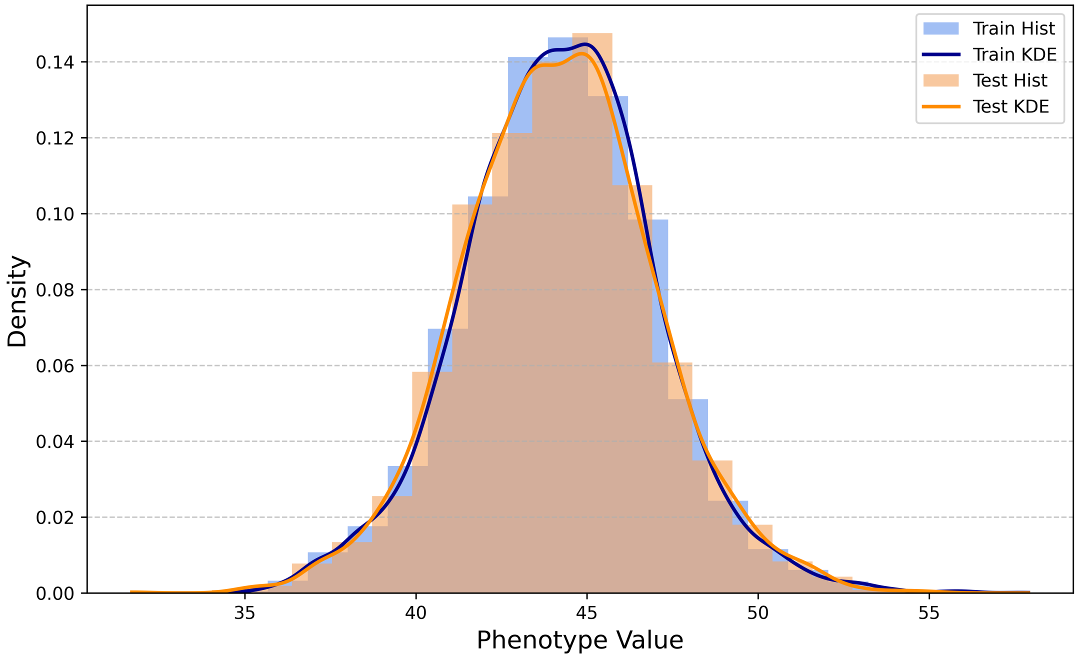

Figure S1. Phenotype distributions for the training and test sets of the soybean *Protein* trait. Histograms and Kernel Density Estimation (KDE) curves compare the training subset with the hold-out test subset to verify distributional consistency.

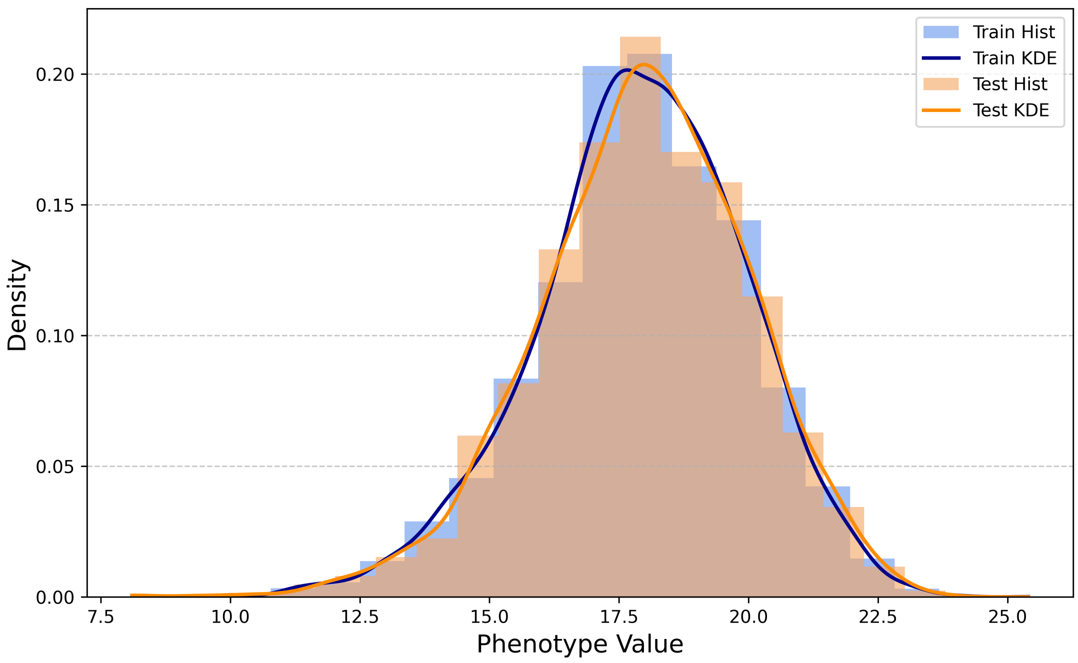

Figure S2. Phenotype distributions for the training and test sets of the soybean *Oil* trait. Histograms and KDE curves are shown for both subsets to confirm consistent phenotype distributions.

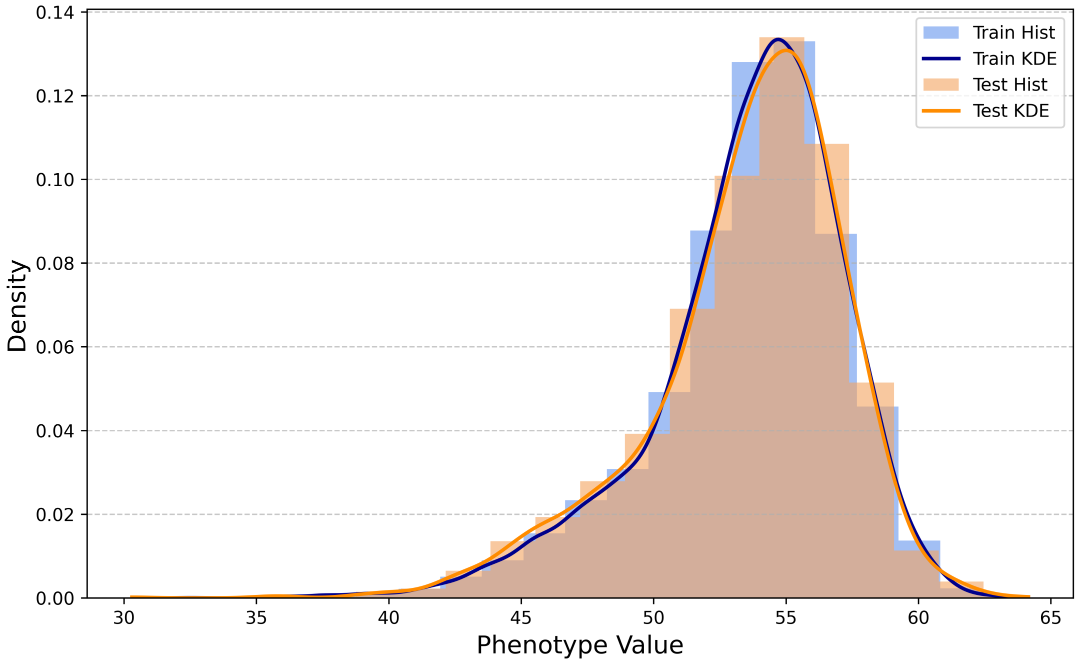

Figure S3. Phenotype distributions for the training and test sets of the soybean *Linoleic* acid content trait. Histograms and KDE curves compare the training and test subsets.

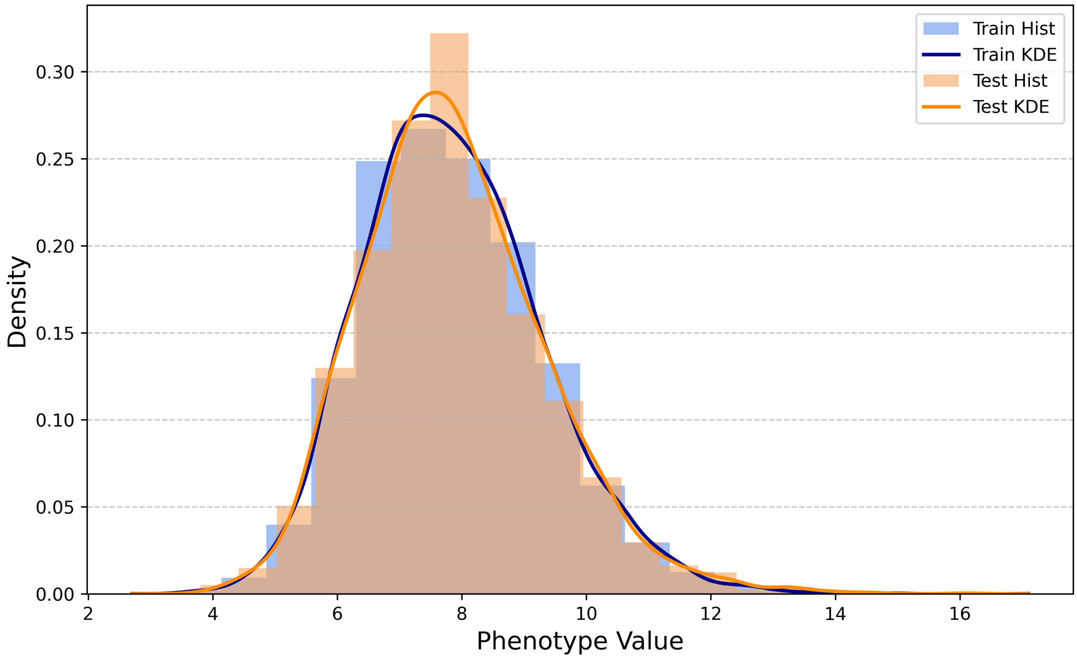

Figure S4. Phenotype distributions for the training and test sets of the soybean *Linolenic* acid content trait. Histograms and KDE curves compare the training and test subsets.

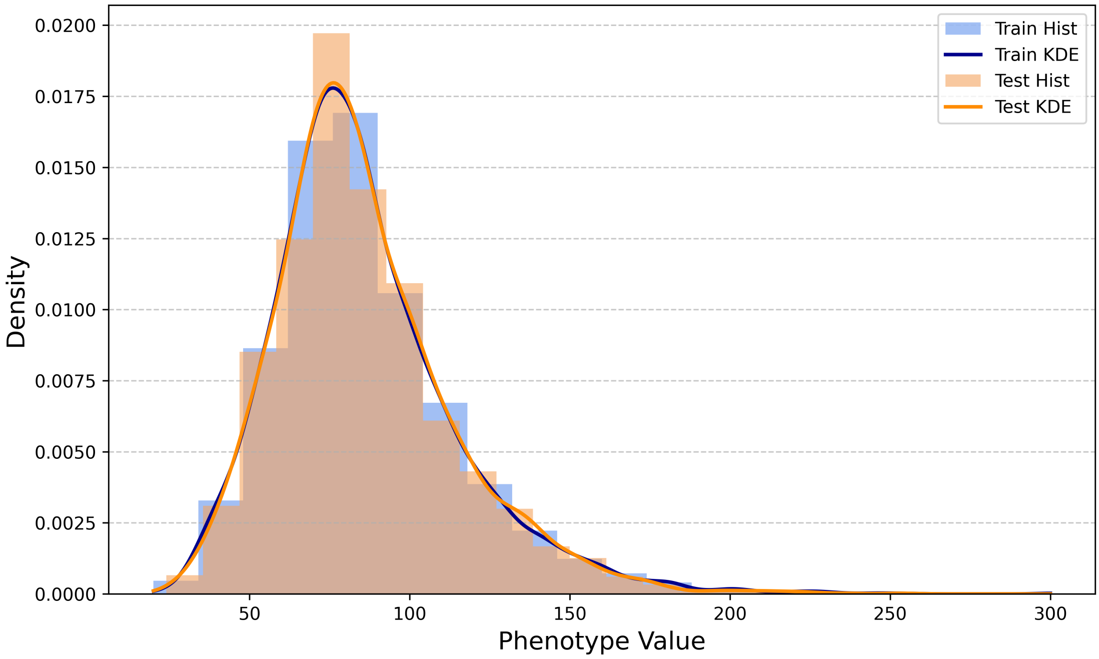

Figure S5. Phenotype distributions for the training and test sets of the soybean *Hgt* (plant height) trait. Histograms and KDE curves compare the training and test subsets.

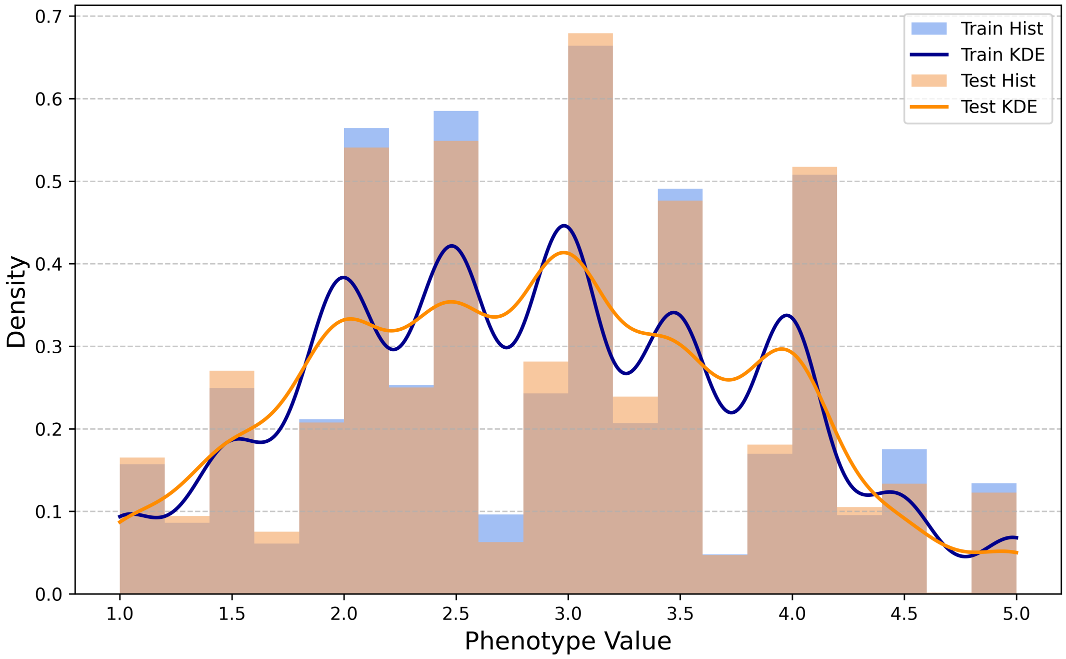

Figure S6. Phenotype distributions for the training and test sets of the soybean *Ldg* (lodging) trait. Histograms and KDE curves compare the training and test subsets.

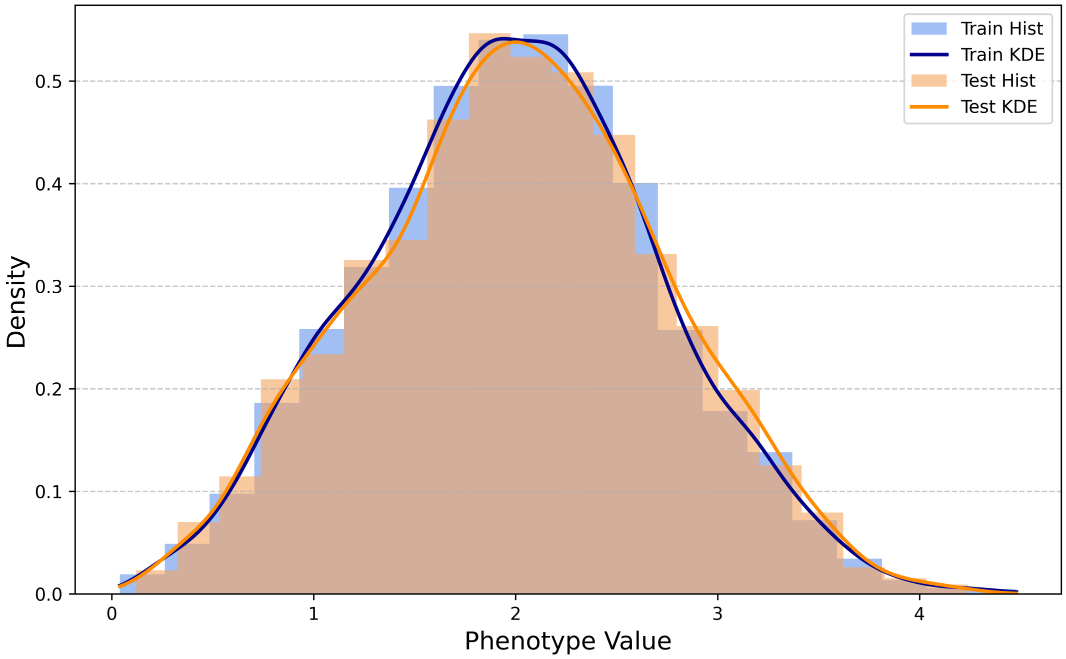

Figure S7. Phenotype distributions for the training and test sets of the soybean *Yield* trait. Histograms and KDE curves compare the training and test subsets.

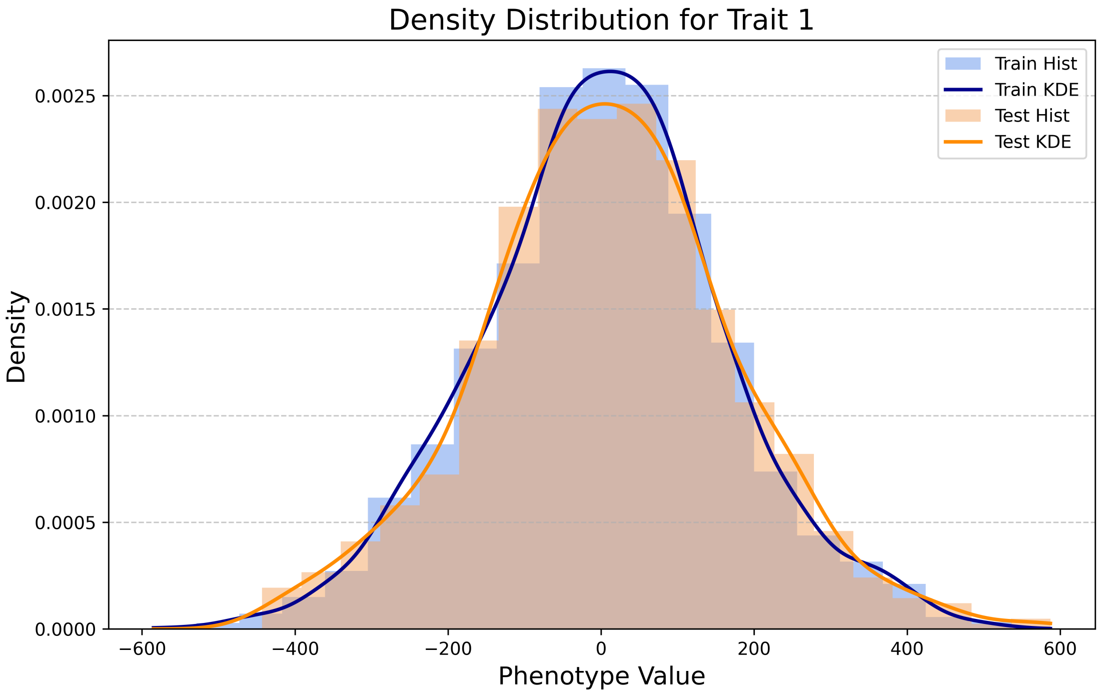

Figure S8. Phenotype distributions for the training and test sets of the simulated dairy cattle *TA* trait. Histograms and KDE curves compare the training and test subsets.

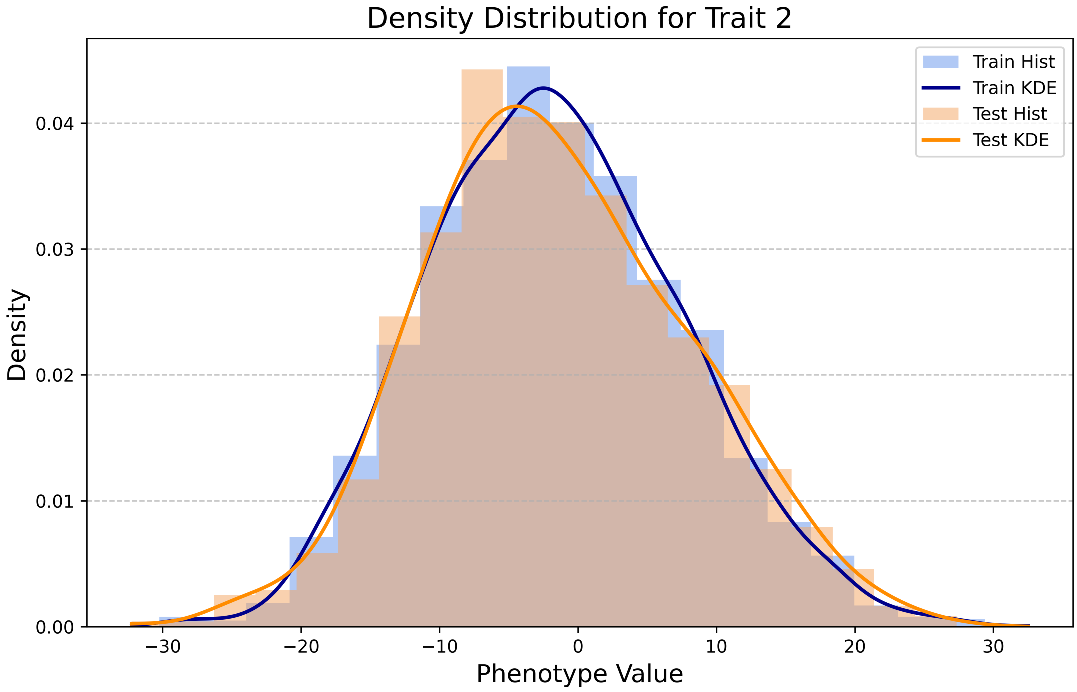

Figure S9. Phenotype distributions for the training and test sets of the simulated dairy cattle *TB* trait. Histograms and KDE curves compare the training and test subsets.

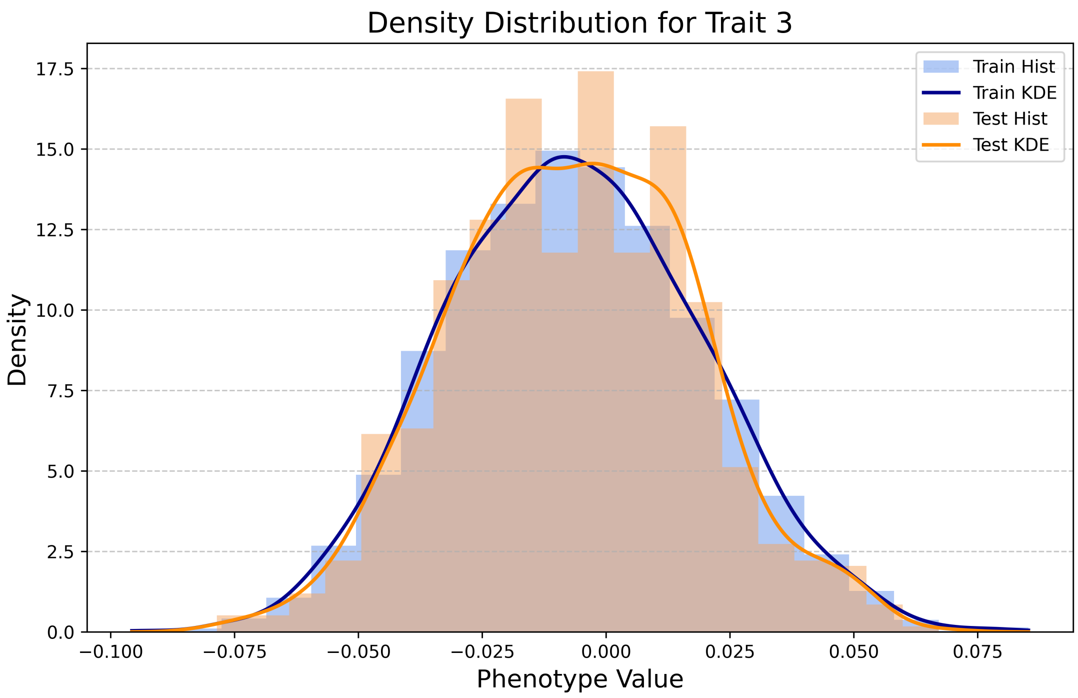

Figure S10. Phenotype distributions for the training and test sets of the simulated dairy cattle *TC* trait. Histograms and KDE curves compare the training and test subsets.

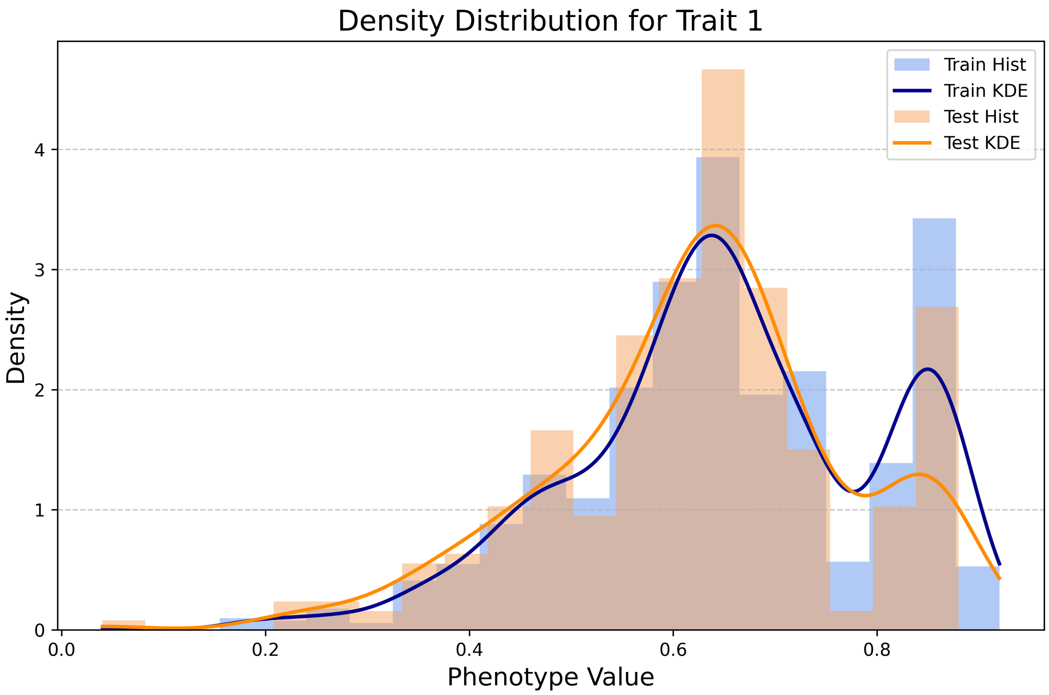

Figure S11. Phenotype distributions for the training and test sets of the Holstein bulls *SM* (sperm motility) trait. Histograms and KDE curves compare the training and test subsets.

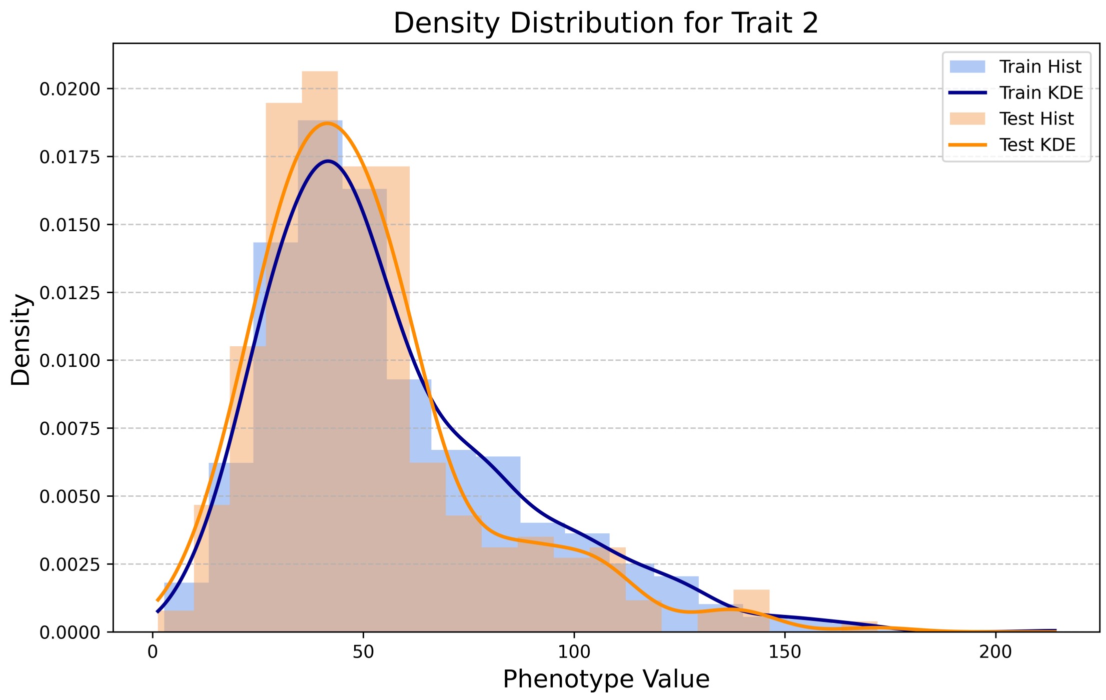

Figure S12. Phenotype distributions for the training and test sets of the Holstein bulls *NMSP* trait. Histograms and KDE curves compare the training and test subsets.

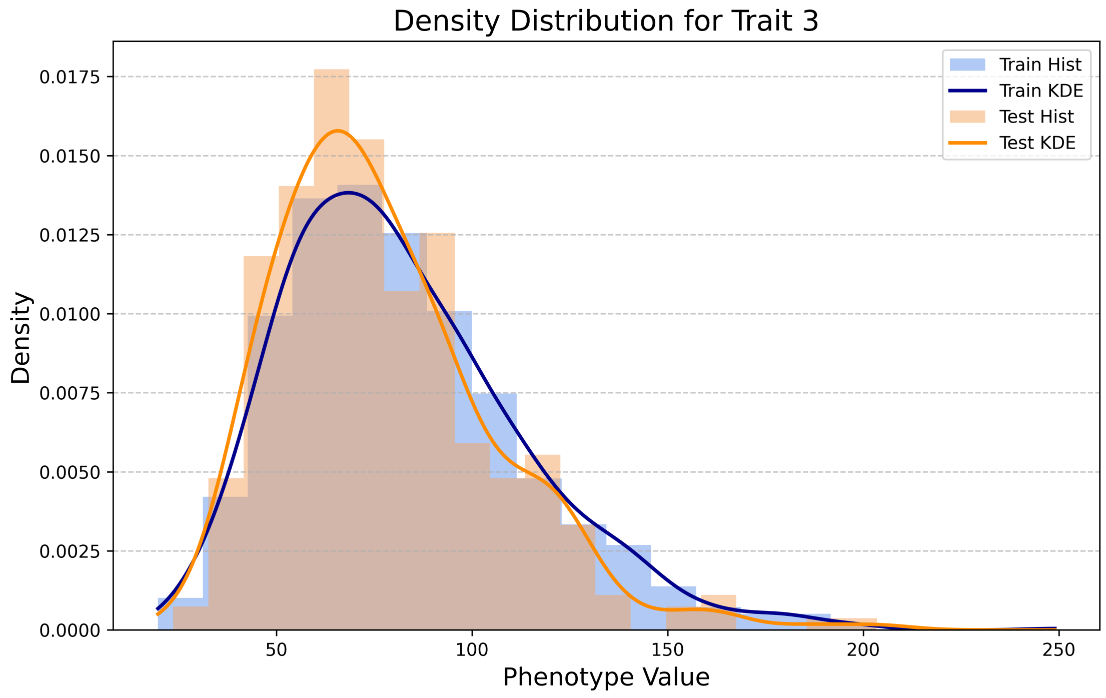

Figure S13. Phenotype distributions for the training and test sets of the Holstein bulls *NSP* trait. Histograms and KDE curves compare the training and test subsets.

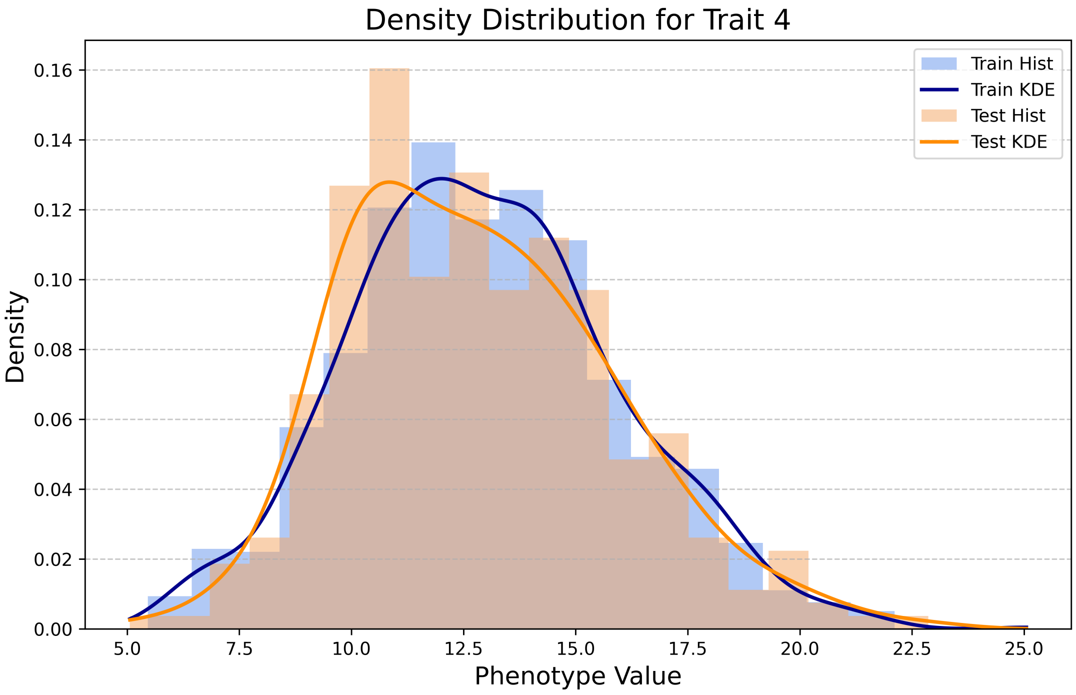

Figure S14. Phenotype distributions for the training and test sets of the Holstein bulls *SC* (sperm concentration) trait. Histograms and KDE curves compare the training and test subsets.

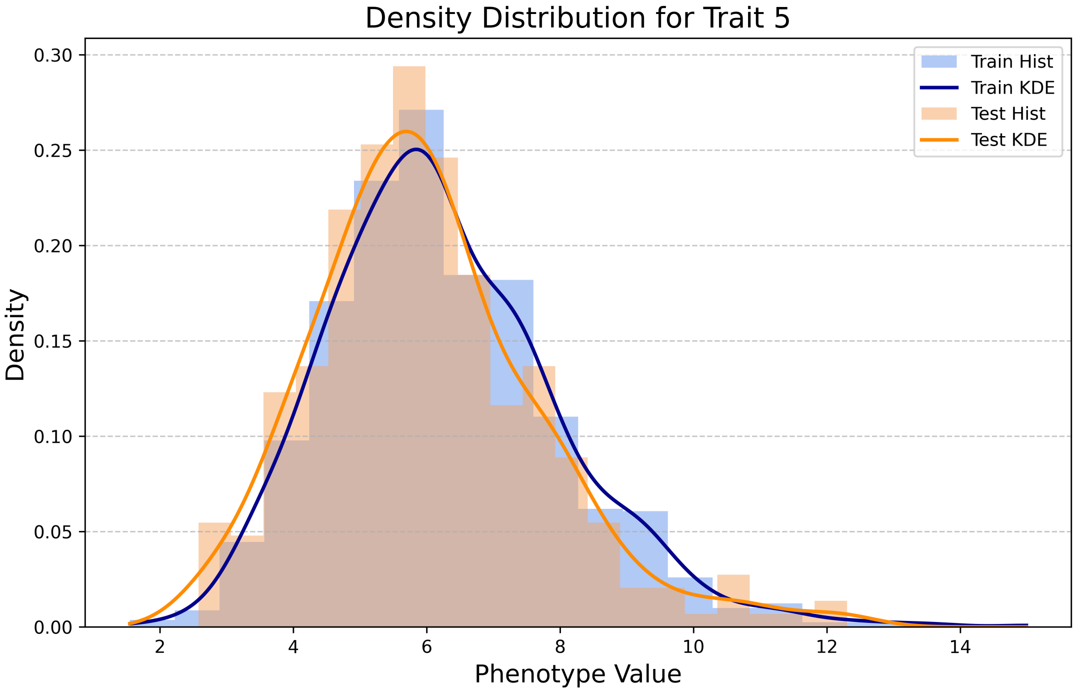

Figure S15. Phenotype distributions for the training and test sets of the Holstein bulls *VE* (ejaculate volume) trait. Histograms and KDE curves compare the training and test subsets.

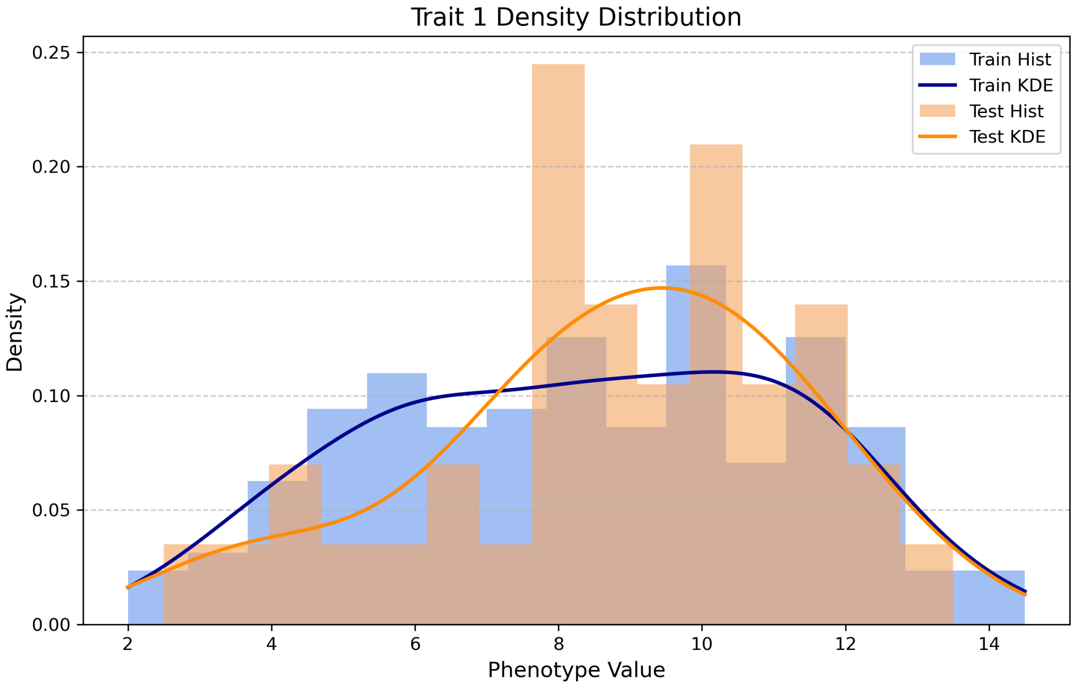

Figure S16. Phenotype distributions for the training and test sets of the chicken *DN* trait. Histograms and KDE curves compare the training and test subsets.

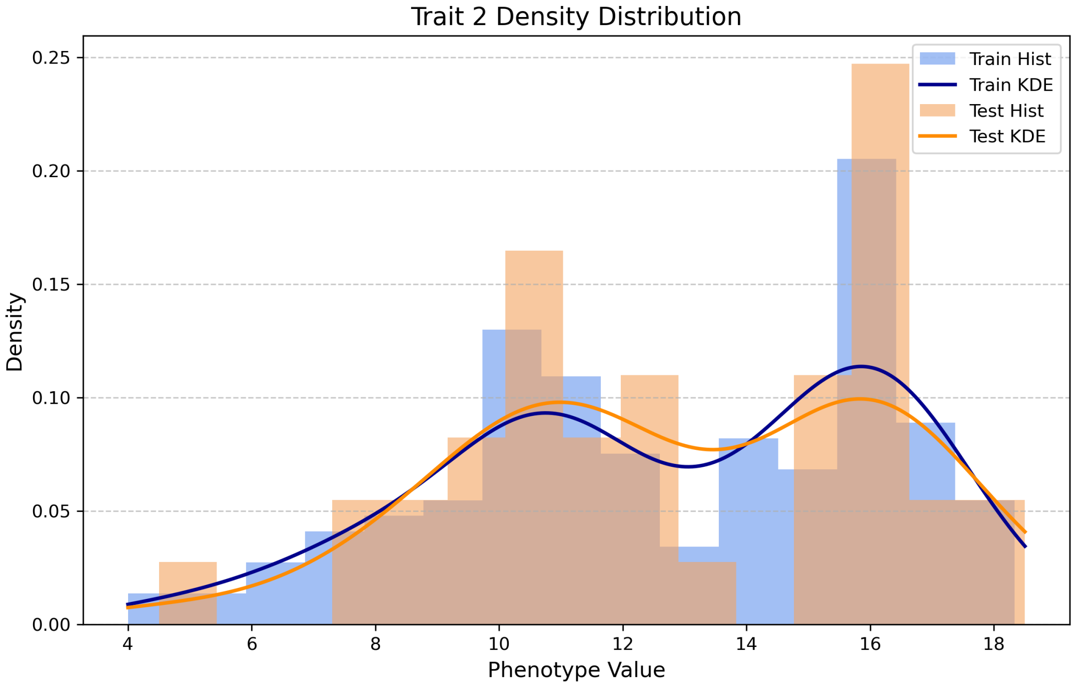

Figure S17. Phenotype distributions for the training and test sets of the chicken *DS* trait. Histograms and KDE curves compare the training and test subsets.

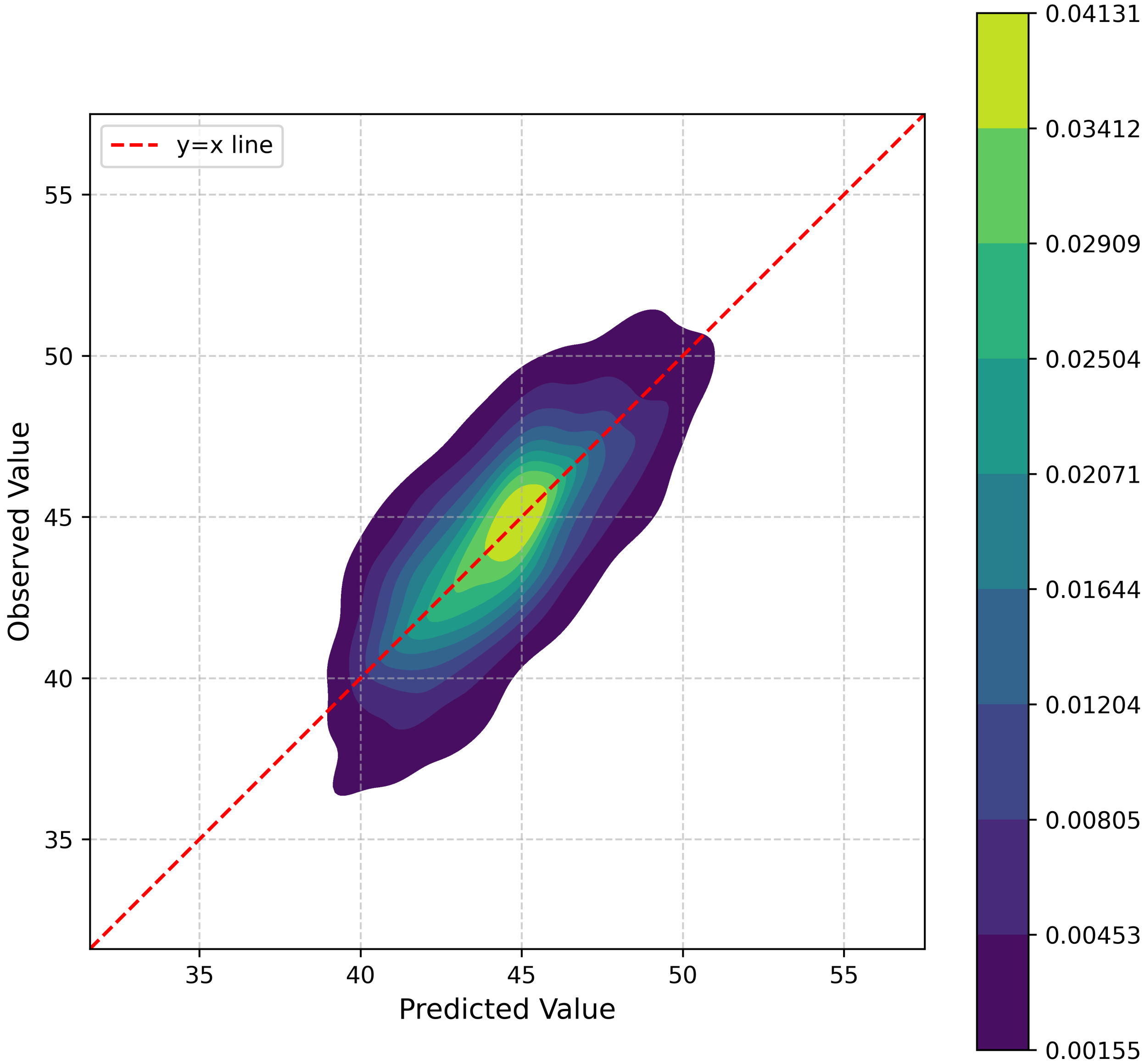

Figure S18. Two-dimensional kernel density plot of predicted versus observed values for the soybean *Protein* trait. The dashed diagonal line indicates perfect prediction ($y = x$); color intensity indicates point density.

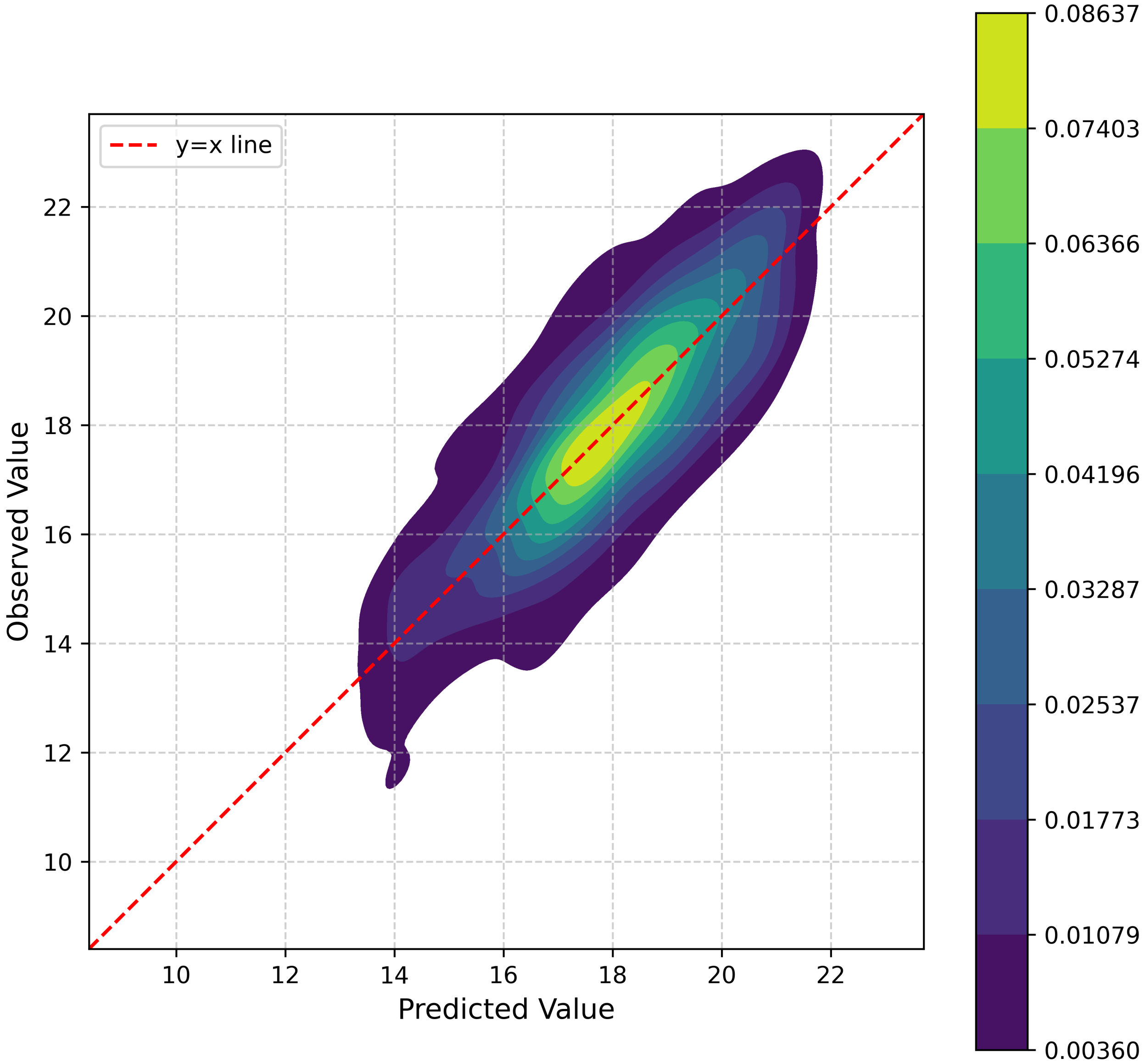

Figure S19. Two-dimensional kernel density plot of predicted versus observed values for the soybean *Oil* trait. The dashed diagonal line indicates perfect prediction ($y = x$); color intensity indicates point density.

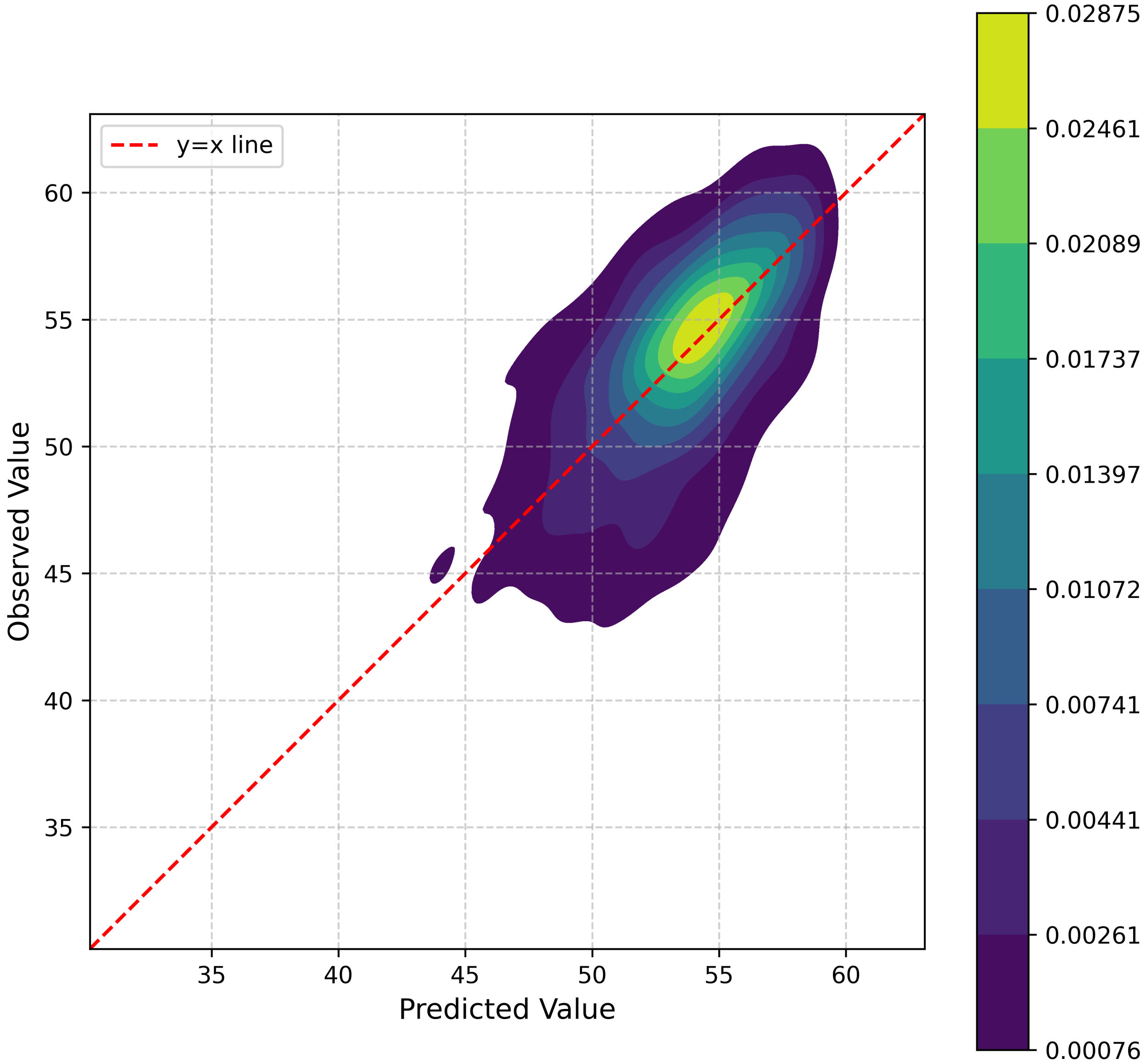

Figure S20. Two-dimensional kernel density plot of predicted versus observed values for the soybean *Linoleic* trait. The dashed diagonal line indicates perfect prediction ($y = x$); color intensity indicates point density.

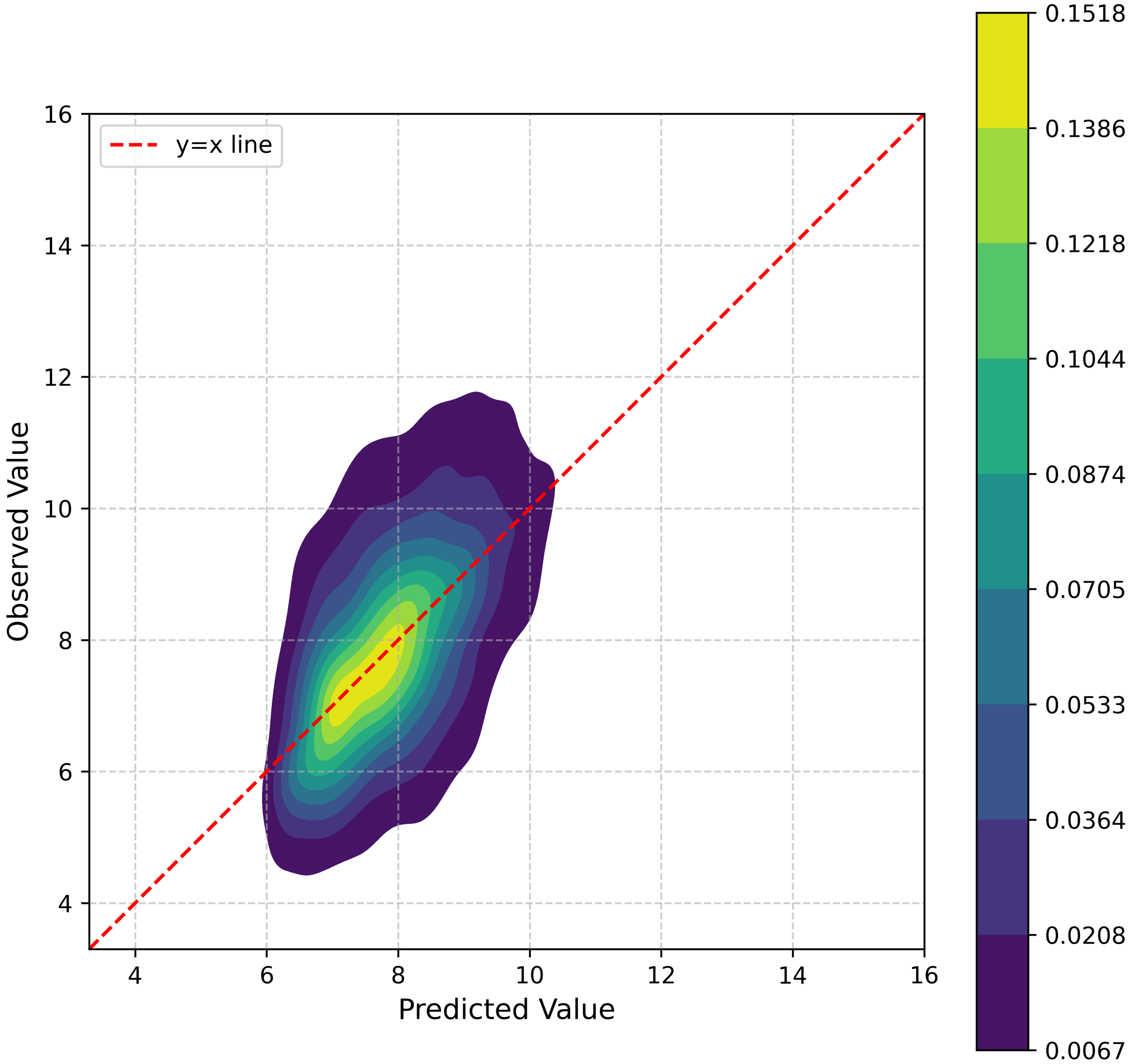

Figure S21. Two-dimensional kernel density plot of predicted versus observed values for the soybean *Linolenic* trait. The dashed diagonal line indicates perfect prediction ($y = x$); color intensity indicates point density.

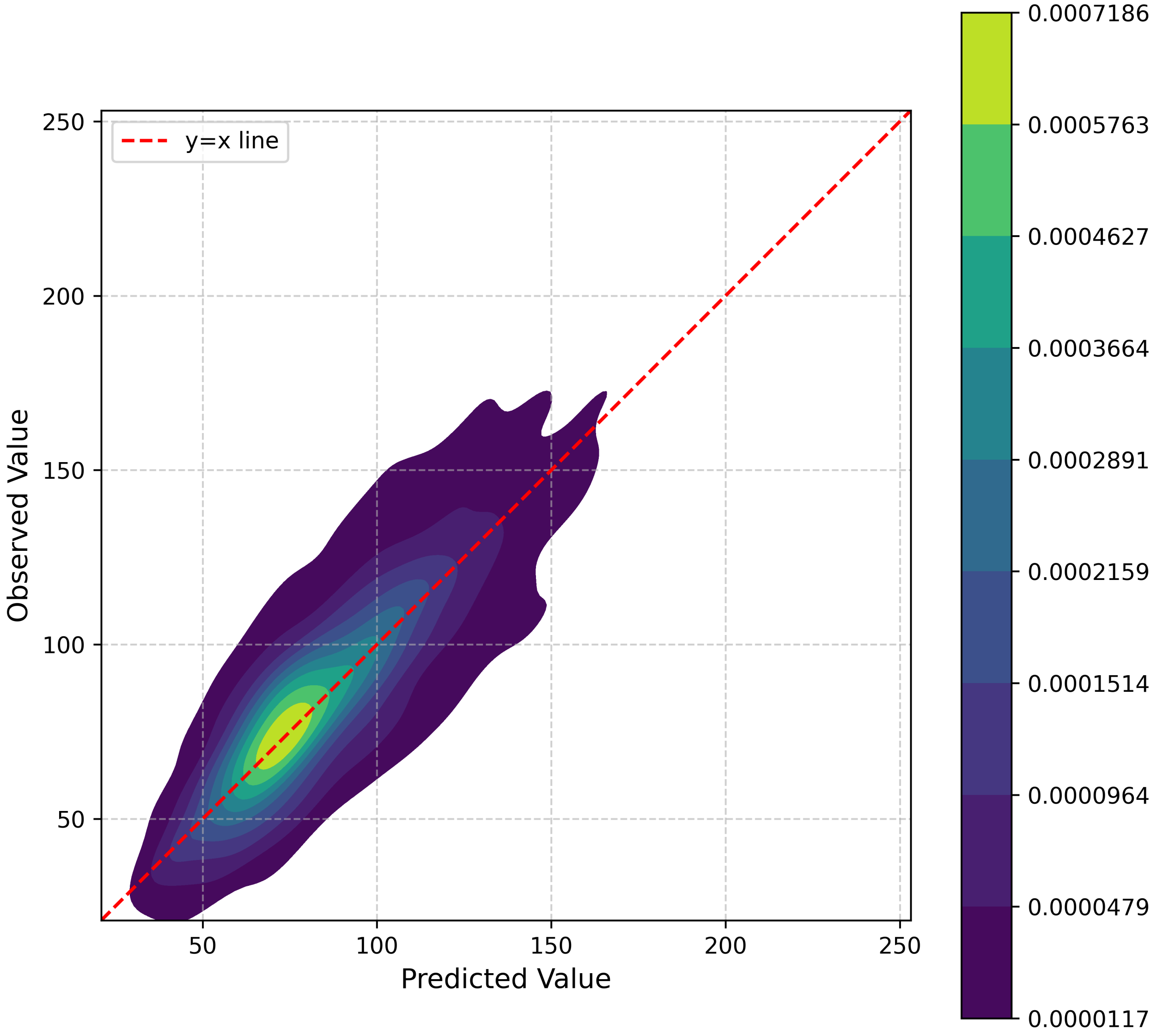

Figure S22. Two-dimensional kernel density plot of predicted versus observed values for the soybean *Hgt* trait. The dashed diagonal line indicates perfect prediction ($y = x$); color intensity indicates point density.

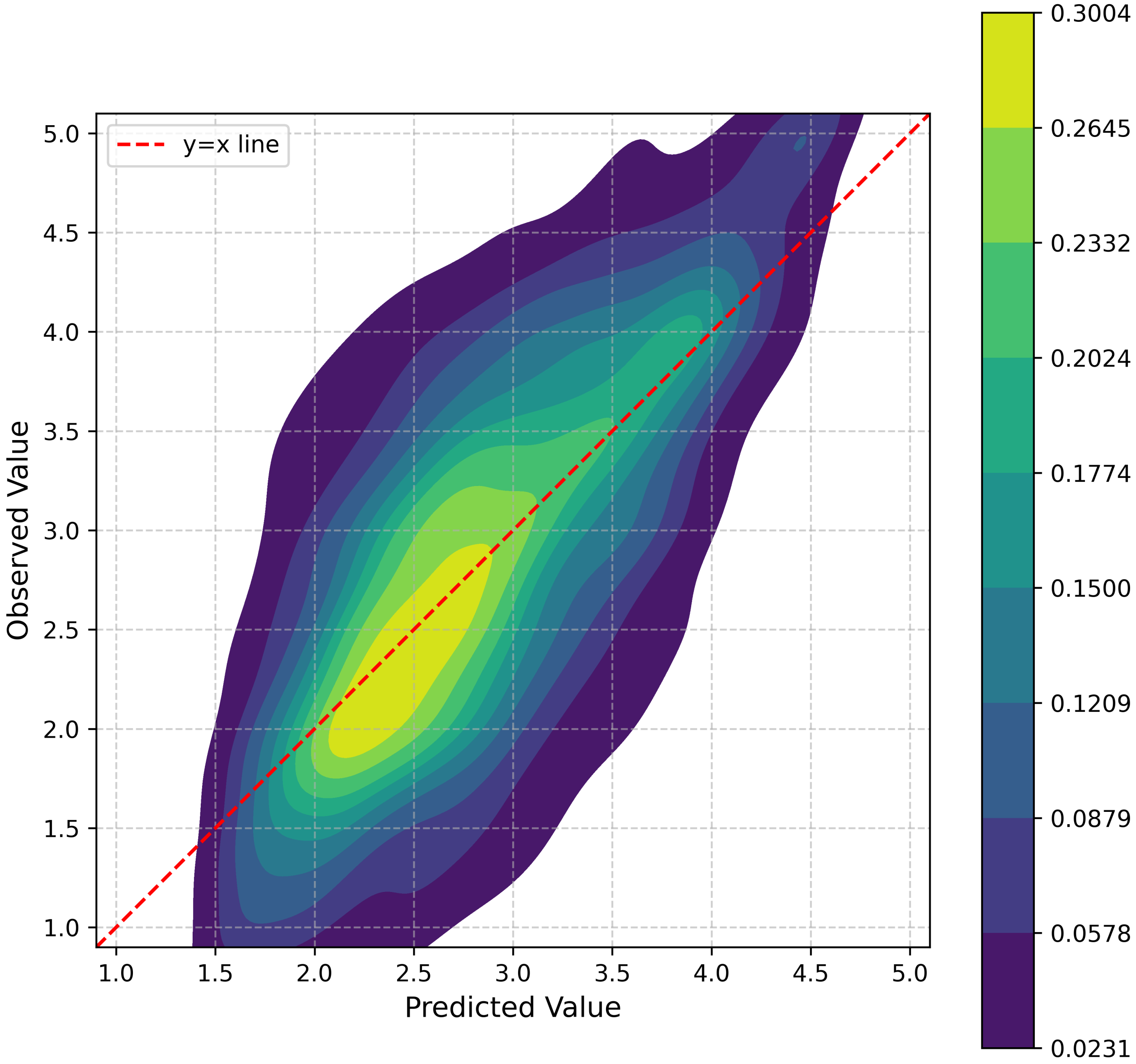

Figure S23. Two-dimensional kernel density plot of predicted versus observed values for the soybean *Ldg* trait. The dashed diagonal line indicates perfect prediction ($y = x$); color intensity indicates point density.

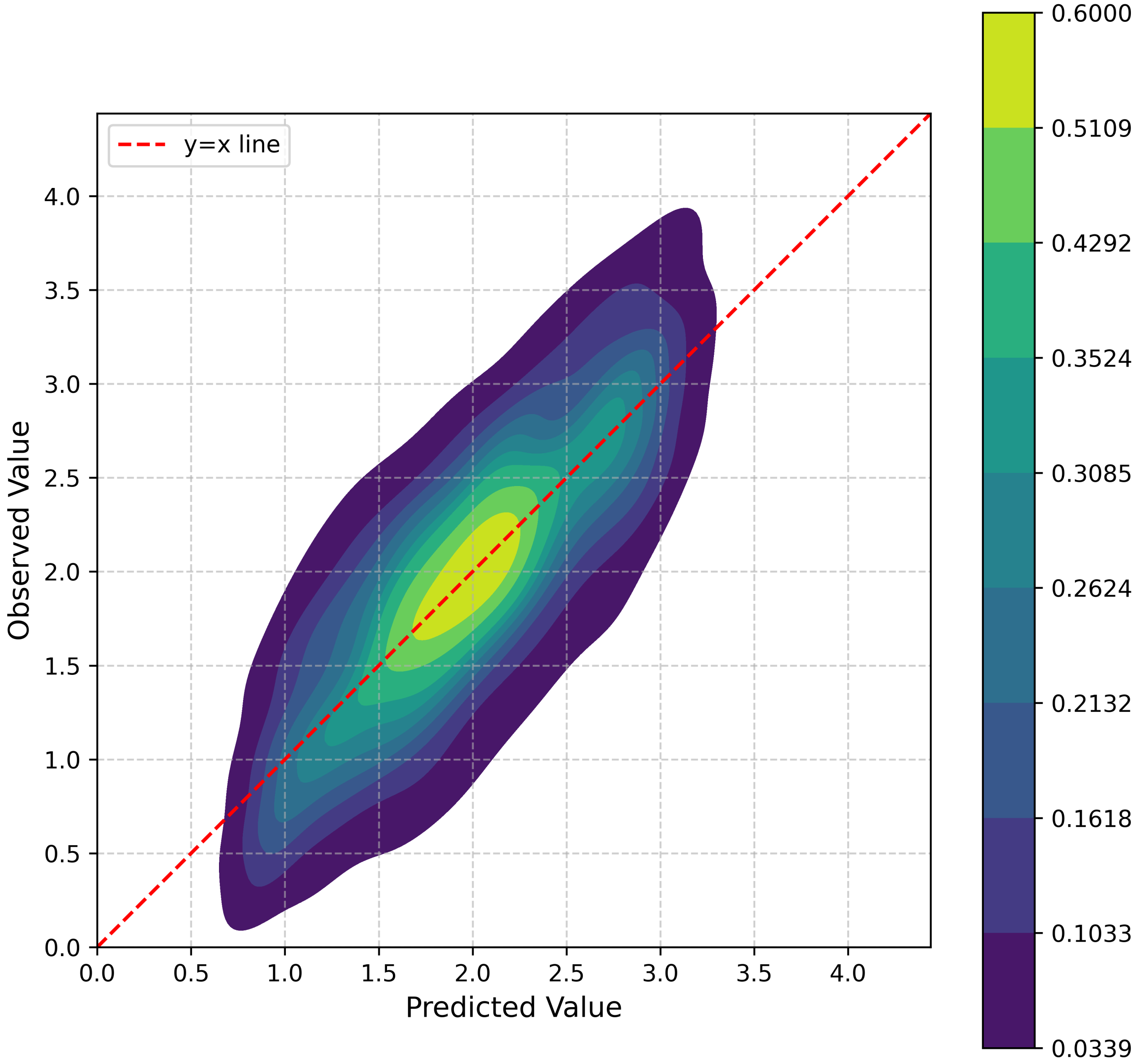

Figure S24. Two-dimensional kernel density plot of predicted versus observed values for the soybean *Yield* trait. The dashed diagonal line indicates perfect prediction ($y = x$); color intensity indicates point density.

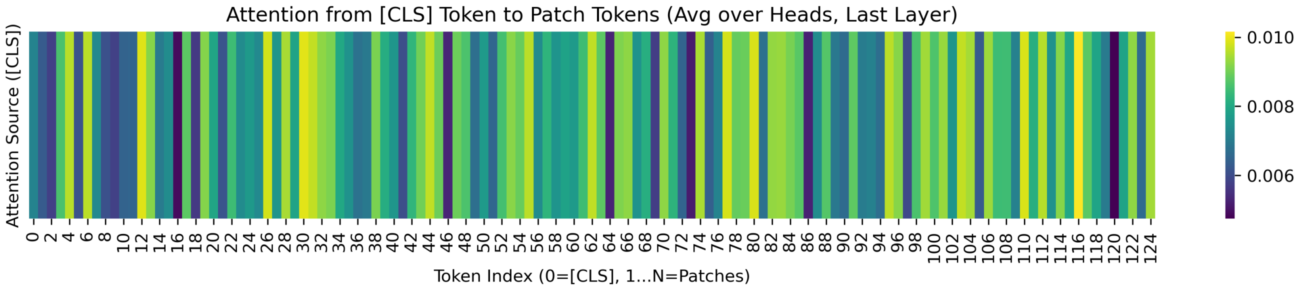

Figure S25. Attention heatmap for the soybean *Protein* trait. The heatmap visualizes non-uniform attention patterns by mapping the [CLS] token attention weights back to genomic coordinates (SNP patches across chromosomes), enabling qualitative inspection of structured signals.

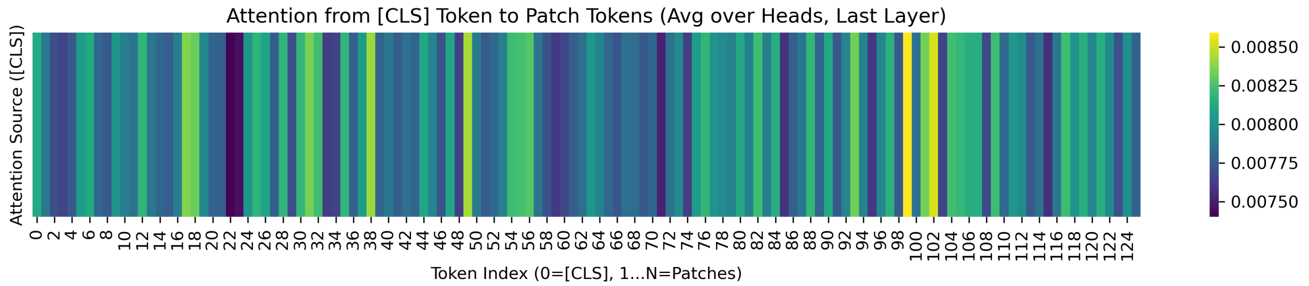

Figure S26. Attention heatmap for the soybean *Linoleic* trait. Attention weights of the [CLS] token are mapped to genomic coordinates for qualitative comparison of structured attention patterns.

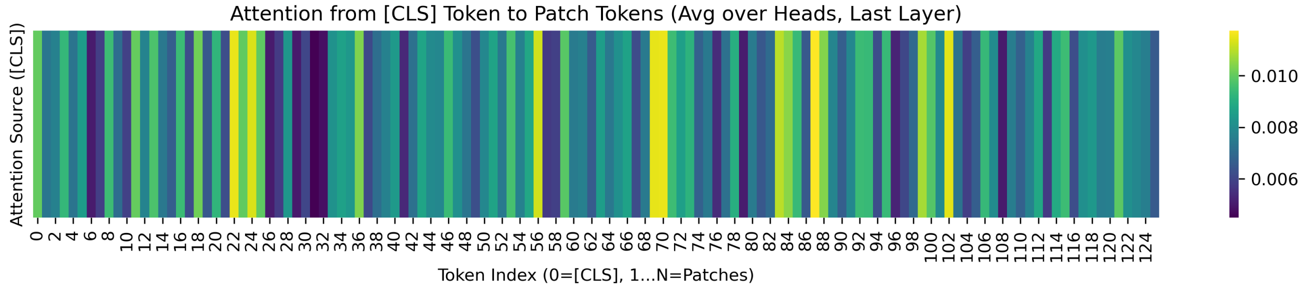

Figure S27. Attention heatmap for the soybean *Linolenic* trait. Attention weights of the [CLS] token are mapped to genomic coordinates for qualitative comparison of structured attention patterns.

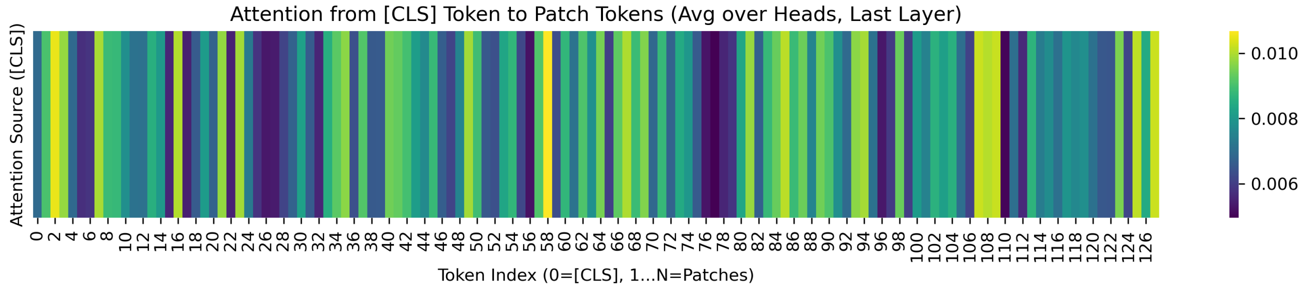

Figure S28. Attention heatmap for the soybean *Hgt* trait. Attention weights of the [CLS] token are mapped to genomic coordinates for qualitative comparison of structured attention patterns.

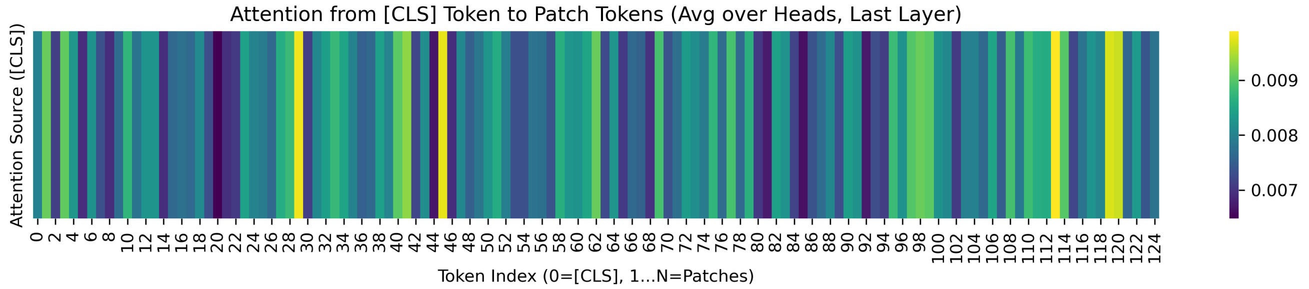

Figure S29. Attention heatmap for the soybean *Mat* trait. Attention weights of the [CLS] token are mapped to genomic coordinates for qualitative comparison of structured attention patterns.

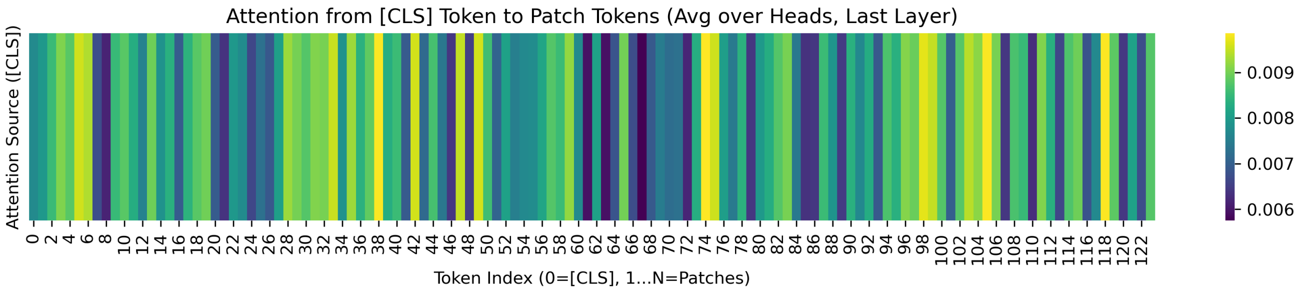

Figure S30. Attention heatmap for the soybean *Ldg* trait. Attention weights of the [CLS] token are mapped to genomic coordinates for qualitative comparison of structured attention patterns.

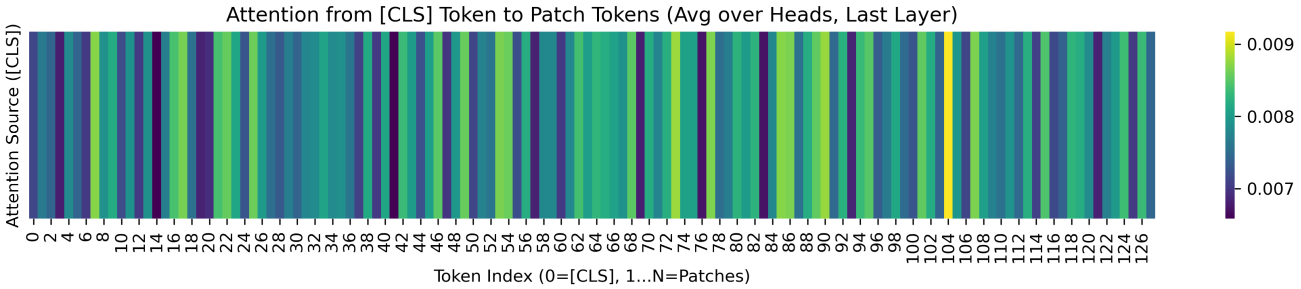

Figure S31. Attention heatmap for the soybean *SdWgt* trait. Attention weights of the [CLS] token are mapped to genomic coordinates for qualitative comparison of structured attention patterns.

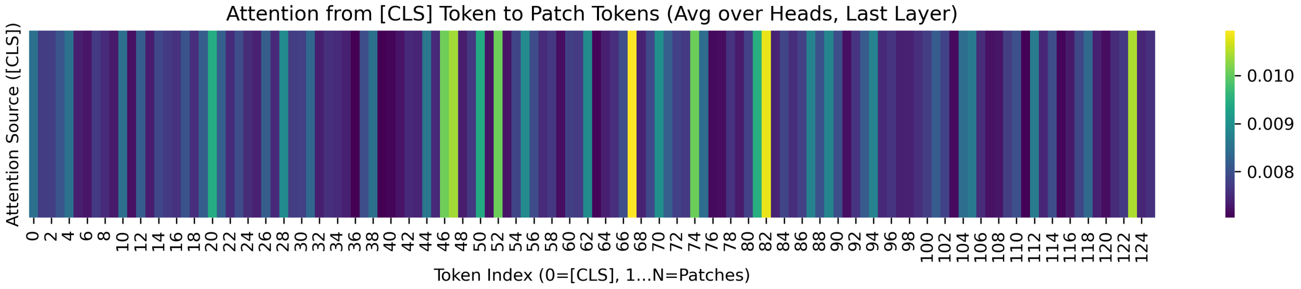

Figure S32. Attention heatmap for the soybean *Yield* trait. Attention weights of the [CLS] token are mapped to genomic coordinates for qualitative comparison of structured attention patterns.

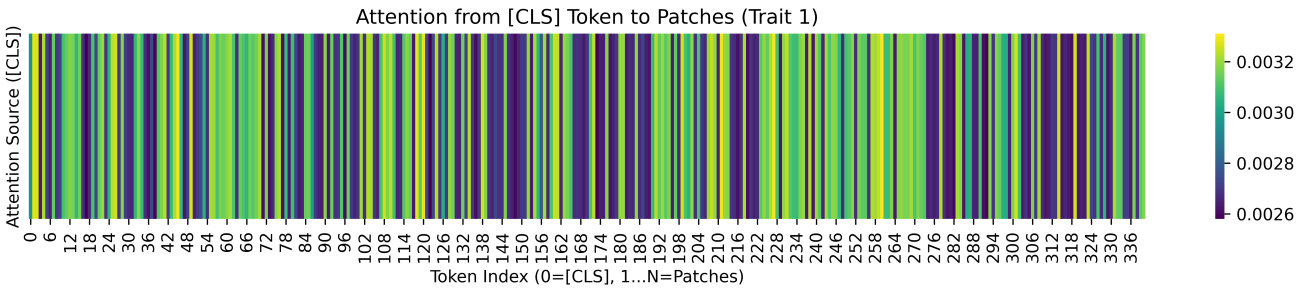

Figure S33. Attention heatmap on the Bulls1508 dataset (example shown for *SM*, sperm motility). The heatmap maps [CLS] attention weights to genomic coordinates (SNP patches) for qualitative comparison of structured, non-uniform attention patterns.

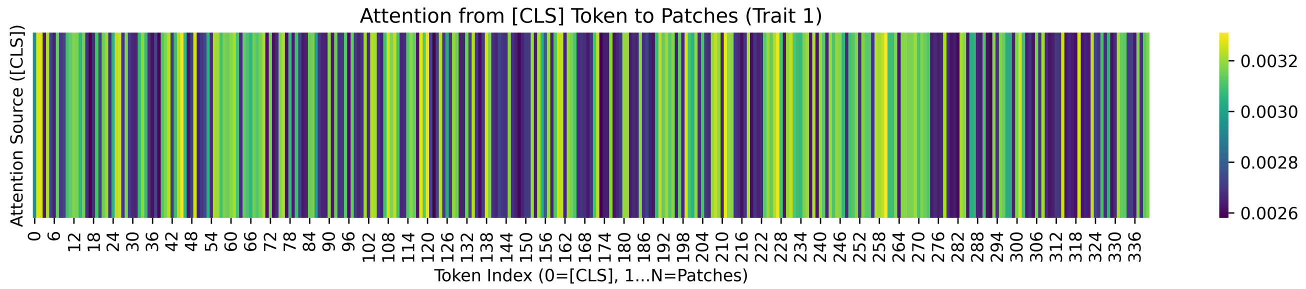

Figure S34. Attention heatmap on the Bulls1508 dataset (example shown for *NSMP*, sperm motility). The heatmap maps [CLS] attention weights to genomic coordinates (SNP patches) for qualitative comparison of structured, non-uniform attention patterns.

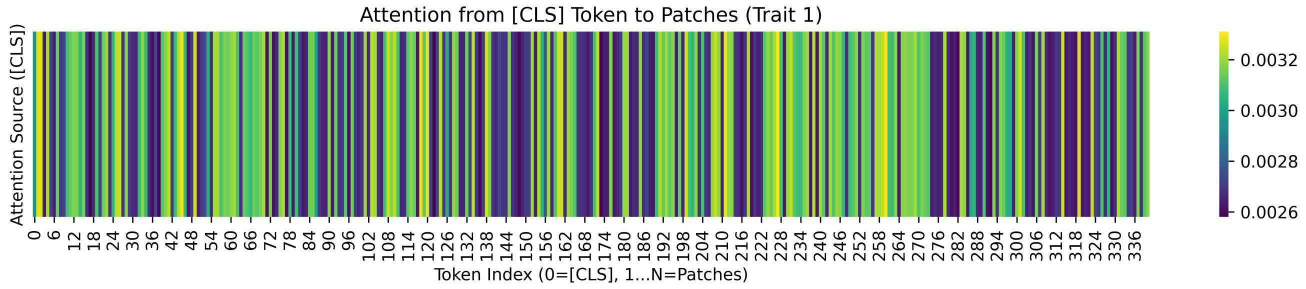

Figure S35. Attention heatmap on the Bulls1508 dataset (example shown for *NSP*, sperm motility). The heatmap maps [CLS] attention weights to genomic coordinates (SNP patches) for qualitative comparison of structured, non-uniform attention patterns.
